# Clinical Outcomes and Evolution of Clonal Hematopoiesis in Patients with Newly Diagnosed Multiple Myeloma

**DOI:** 10.1158/2767-9764.CRC-23-0093

**Published:** 2023-12-18

**Authors:** Tarek H. Mouhieddine, Chidimma Nzerem, Robert Redd, Andrew Dunford, Matthew Leventhal, Romanos Sklavenitis-Pistofidis, Sabrin Tahri, Habib El-Khoury, David P. Steensma, Benjamin L. Ebert, Robert J. Soiffer, Jonathan J. Keats, Shaadi Mehr, Daniel Auclair, Irene M. Ghobrial, Adam S. Sperling, Chip Stewart, Gad Getz

**Affiliations:** 1 Department of Medical Oncology, Dana-Farber Cancer Institute, Boston, Massachusetts.; 2 Harvard Medical School, Boston, Massachusetts.; 3 Broad Institute of MIT and Harvard, Cambridge, Massachusetts.; 4 Division of Hematology and Medical Oncology, Tisch Cancer Institute, Icahn School of Medicine at Mount Sinai, New York, New York.; 5 Harvard T.H. Chan School of Public Health, Boston, Massachusetts.; 6 Department of Data Sciences, Dana-Farber Cancer Institute, Boston, Massachusetts.; 7 Department of Hematology, Erasmus MC Cancer Centre, Rotterdam, the Netherlands.; 8 Integrated Cancer Genomics Division, Translational Genomics Research Institute, Phoenix, Arizona.; 9 Multiple Myeloma Research Foundation, Norwalk, Connecticut.; 10 Division of Hematology, Brigham and Women's Hospital, Boston, Massachusetts.; 11 Cancer Center and Department of Pathology, Massachusetts General Hospital, Boston, Massachusetts.

## Abstract

Clonal hematopoiesis (CH) at time of autologous stem cell transplant (ASCT) has been shown to be associated with decreased overall survival (OS) and progression-free survival (PFS) in patients with multiple myeloma not receiving immunomodulatory drugs (IMiD). However, the significance of CH in newly diagnosed patients, including transplant ineligible patients, and its effect on clonal evolution during multiple myeloma therapy in the era of novel agents, has not been well studied. Using our new algorithm to differentiate tumor and germline mutations from CH, we detected CH in approximately 10% of 986 patients with multiple myeloma from the **C**linical **O**utcomes in **MM** to **P**ersonal **Ass**essment of Genetic Profile (CoMMpass) cohort (40/529 transplanted and 59/457 non-transplanted patients). CH was associated with increased age, risk of recurrent bacterial infections and cardiovascular disease. CH at time of multiple myeloma diagnosis was not associated with inferior OS or PFS regardless of undergoing ASCT, and all patients benefited from IMiD-based therapies, irrespective of the presence of CH. Serial sampling of 52 patients revealed the emergence of CH over a median of 3 years of treatment, increasing its prevalence to 25%, mostly with *DNMT3A* mutations.

**Significance:**

Using our algorithm to differentiate tumor and germline mutations from CH mutations, we detected CH in approximately 10% of patients with newly diagnosed myeloma, including both transplant eligible and ineligible patients. Receiving IMiDs improved outcomes irrespective of CH status, but the prevalence of CH significantly rose throughout myeloma-directed therapy.

## Introduction

Multiple myeloma is an incurable plasma cell malignancy that evolves from the precursor disorders monoclonal gammopathy of undetermined significance and smoldering multiple myeloma (1–3). The standard of care for patients with newly diagnosed multiple myeloma is induction therapy with a three- or four-drug combination therapy followed by high-dose melphalan and autologous stem cell transplant (ASCT) and indefinite maintenance therapy, most commonly with an immunomodulatory drug (IMiD). Older patients or those with comorbid conditions that make them unfit for ASCT, typically undergo multi-agent induction followed by maintenance therapy (2). Furthermore, multiple studies have identified both clinical and genomic factors that contribute to a faster rate of progression from precursors to overt myeloma (4–9), shorter progression-free survival (PFS) and decreased overall survival (OS) in patients with multiple myeloma undergoing treatment (1, 10–12). However, many patients demonstrate PFS or OS that is significantly shorter than expected from these models, and it is clear that additional factors that influence treatment response and disease aggressiveness have yet to be described.

Clonal hematopoiesis (CH) refers to the presence of a population of expanded, somatically mutated, hematopoietic stem cells (HSC) that can be detected in the peripheral blood (PB; ref. 13). The mutations in CH are often found in genes recurrently mutated in hematologic malignancies and are believed to provide a fitness advantage to the mutant HSCs. The presence of CH is associated with increased risk of hematologic malignancies (14, 15), namely myelodysplastic syndrome (MDS) and acute myeloid leukemia (AML; refs. 16, 17). The risk of CH is associated with older age, prior smoking, and history of exposure to radiation and cytotoxic chemotherapy (18–21). In addition to hematologic malignancies, patients with CH are at higher risk of inflammatory conditions including cardiovascular disease (22), chronic obstructive pulmonary disease (23), and gout (24), among other conditions, which are at least in part thought to be mediated by altered inflammatory signaling in mutant macrophages (22).

We recently reported that approximately 21.6% of patients with multiple myeloma have coincident CH with mutations at a variant allele fraction (VAF) of at least 1% at the time of ASCT. In this cohort, the presence of CH was associated with shorter OS and PFS, particularly in those who did not receive maintenance therapy with an IMiD (25). These findings suggest a possible interaction between multiple myeloma cells and the somatically mutated stem and myeloid cells in the tumor microenvironment. While intriguing, this finding was based on data from a single tertiary cancer center, and only included patients with multiple myeloma who received ASCT; moreover, all samples were collected at the time of ASCT, after the initiation of therapy. Serial samples were not available for analysis to evaluate evolution of CH during multiple myeloma therapy. To further assess the association between CH and clinical outcomes in patients with newly diagnosed multiple myeloma, we developed a novel Bayesian method to differentiate CH mutations from germline and somatic tumor mutations and used it to study a large multi-center cohort of 986 newly diagnosed multiple myeloma cases, including transplant eligible and ineligible patients. Serial sampling in a subset of patients also allowed us to investigate the temporal dynamics of CH clones.

## Materials and Methods

### Multiple Myeloma Research Foundation Cohort

The Multiple Myeloma Research Foundation (MMRF) cohort is composed of 986 patients with newly diagnosed multiple myeloma whose PB and bone marrow (BM) samples were collected starting July 2011 up until the present time. All patients provided written informed consent to allow the collection and clinical and genetic analysis of PB and BM samples for research purposes. The study design complied with the Declaration of Helsinki and the International Conference on Harmonization Guidelines for Good Clinical Practice. Whole-exome sequencing (WES) data of PB and BM samples of 986 patients with multiple myeloma (529 transplanted and 457 non-transplanted) were downloaded from the Multiple Myeloma Research Consortium **C**linical **O**utcomes in **MM** to **P**ersonal **Ass**essment of Genetic Profile (CoMMpass, NCT0145429) study (26) in the database of Genotype and Phenotype (# phs000748.v6.p4) and their PB data were analyzed for mutations in CH-associated genes (Supplementary Table S1). Clinical data (MMRF IA18 dataset) were downloaded from the MMRF web portal (https://research.themmrf.org/). Targeted sequencing data were also acquired on 52 patients, for which sequential PB samples were available. For targeted sequencing, a bait panel of 568 genes was used, including pan-cancer and myeloid-associated genes (Supplementary Table S2). All MMRF samples underwent next-generation sequencing at the Translational Genomics Research Institute (TGen), Phoenix, AZ.

### Detection of Candidate CH Mutations

The first step in the analysis was to detect candidate CH mutations. Sequencing data were aligned using BWA-mem and the base qualities of the aligned data were recalibrated using GATK3 Base Quality Score Recalibration (BQSR; refs. 27, 28). The WES samples had an average coverage of 113X across samples (range 14X-257X). Next, we ran a modified version of the Getz Lab CGA WES Characterization pipeline (https://portal.firecloud.org/#methods/getzlab/CGA_WES_Characterization_Pipeline_v0.2_Jun2019/5) to call, filter, and annotate somatic mutations and copy-number variations (CNV). We modified this pipeline to call blood samples without matched BM biopsy samples by using a single PB sample (MMRF_1474_1_PB) of the youngest patient who was a 27-year-old male, that had no CH mutations, as a pseudogermline control for all PB samples. We used MuTect1 (29) for single-nucleotide variant (SNV); Strelka (30) and GATK MuTect2 (31) to call indels; Orientation Bias Filter (32), MAFPonFilter (33), and RealignmentFilter to filter technical artifacts; ABSOLUTE (34) to estimate clonality; PicardTools (ref. 31; https://software.broadinstitute.org/gatk/documentation/tooldocs/4.0.0.0/picard_analysis_CollectMultipleMetrics.php) for quality control metrics; and Variant Effect Predictor (35) and Oncotator (36) to annotate variants (see Supplementary Materials and Methods). All variant detection for this cohort was based on the pipeline described previously (25), which included a preliminary selection of CH candidate mutations based on AML drivers that served as a catalogue of potential CH mutations (Supplementary Table S1).

To estimate contamination with DNA from other individuals, we used VerifyBamID (37) using the ExAC (38) VCF to test for germline SNPs with a minimum allele frequency of 25% in the GnomAD population. To control for noise with indel calling, we used the PB sample MMRF_1474_1_PB of the youngest patient (27 years old) with no detectable CH mutations to use as an unmatched control for Strelka and MuTect2. We focused only on variants that were classified as pathogenic or likely pathogenic CH mutations based on mutation type, position, and frequency in published reports (14, 19, 39) and public databases (40). The set of rules to include candidate CH mutations is outlined in Supplementary Table S2. We filtered out variant calls if they had 3 or fewer supporting reads. Initial selection criteria for qualifying variants included having a VAF of ≥2% followed by a processing step in which evidence for each candidate mutation was re-evaluated by MutationValidator (https://portal.firecloud.org/#methods/broadinstitute_cga/mutation_validator/11) and manual review using Integrated Genome Viewer (IGV; ref. 41). MutationValidator's allele counting identified one candidate CH mutation in *TET2* with a VAF less than 2%, but we retained this event in the analysis cohort because it passed the initial selection criteria. Furthermore, except for *DNMT3A, TET2, ASXL1, PPM1D, TP53, JAK2, SF3B1,* and *SRSF2*, mutations with VAF above 35% were also excluded because these often represent germline polymorphisms. Then, we removed technical artifacts (Supplementary Materials and Methods). Note that the BM samples were first sorted to enrich for myeloma cells prior to WES, thus making our BM sample purities relatively high (median of 93%, range 1%–100%). Overall, this analysis yielded 151 candidate CH variants across 129 patients.

### Mutation Classification to CH, Tumor, or Germline

The next step in the analysis was to test each candidate CH mutation as a possible germline variant or BM tumor mutation by comparison of the allele counts observed in the PB sample and the matched BM sample (Supplementary Fig. S1). We used a Bayesian approach to classify mutations: We combined prior knowledge about mutation frequencies (expressed as prior probabilities) with observed allele counts (expressed as likelihood functions) to generate three posterior distribution models for the origins of the variants: (i) a true CH mutation, (ii) a somatic tumor mutation, and (iii) a germline variant. We note that because we observe a discrete number of alternate (*alt*) and reference (*ref*) reads for each variant, we do not know the true underlying VAF but rather use a Beta distribution to represent the likelihood of the VAF (θ), given the allele counts:



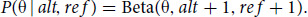



The most likely underlying VAF, 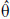, is the commonly used estimate 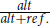


Next, we considered that cross-contamination of the PB and BM samples can occur (Supplementary Fig. S2). We used deTiN (42) to calculate tumor-in-normal (TiN) contamination of PB samples. We used ABSOLUTE (34) to determine (i) the fraction of cancer cells in each BM sample, typically called purity (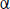), (ii) the average DNA per tumor cell (in units of haploid genomes) in each BM sample, typically called ploidy (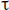), and (iii) the local absolute copy number in the tumor cells at the mutated locus.

Under the germline model, we do not always expect a VAF of 0.5 in a BM sample due to potential CNVs in tumor cells. Because of possible two-way contamination of PB and BM samples, that is, tumor DNA in the PB sample and blood (part of the non-tumor DNA) in the BM sample, we express the germline VAF in each PB sample as:



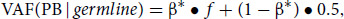



and the germline VAF in each BM sample as:



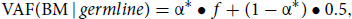



where 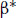 is the site-specific fraction of tumor DNA in the PB sample (which we derive from the TiN values; Supplementary Materials and Methods), 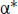 is the site-specific fraction of tumor DNA in the BM sample (which we derive from the tumor purity 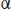, Supplementary Materials and Methods), and *f* is the site-specific heterozygous germline allelic copy ratio in the tumor cells. There are two possible values of *f* (one for each parental allele) and we choose the value closest to the observed VAF as the most likely (Supplementary Materials and Methods).

Under the tumor somatic mutation model, the expected VAF in each PB sample due to two-way contamination can be expressed as follows:



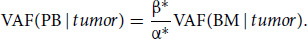



Similarly, a BM sample often contains some fraction of DNA from non-cancer cells, some of which may originate from the CH clone. Therefore, reads supporting a CH mutation may be observed in the BM sequencing data. Because both the BM and PB samples can have blood and, therefore, the CH clone in them, under a CH mutation model, we express the expected VAF in the BM sample as follows:



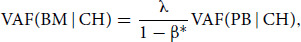



where 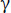 is the fraction of PB DNA in the tumor sample, which represents part of the non-cancer DNA in the BM sample. Therefore, 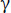 is at most 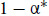, if all the non-cancer contribution to the BM samples comes from PB, and in general, 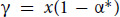 where *x*, between 0 and 1, reflects the unknown fraction of non-cancer DNA that originates from the PB (because we do not know *x*, we integrate over its possible values in the model; Supplementary Materials and Methods).

The likelihoods are described by the following joint models:



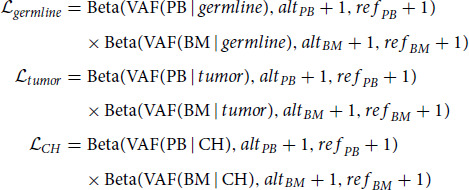



where 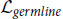, 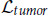, and 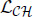 are joint likelihoods of a variant originating under the hypotheses of germline, tumor, and CH; and 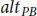, 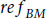, 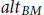, and 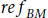 are the observed alternate and reference allele counts for PB and BM samples. Note that these models do not assume the clonality of the CH or tumor mutations.

As a final step in the classification of mutations, we multiply the prior probabilities by the likelihood values to generate posterior probabilities of CH, tumor, and germline origin for every mutation. For each mutation, the final classification corresponds to the hypothesis with the largest posterior probability.

Next, to study the dynamics of CH mutations over time, we performed the same analysis on 20 additional candidate CH mutations identified in sequential PB samples taken from the patients who relapsed while on treatment. This analysis also enabled us to compare the classification of the mutations at different timepoints. Reassuringly, all 20 candidate CH mutations were consistently classified in the first and subsequent timepoints, with 14/20 classified as CH, 3/20 as tumor, and the remaining 3 as germline.

### Statistical Analyses

OS was defined as the time from the date of myeloma diagnosis until death from any cause, with censoring at the date last known to be alive. PFS was measured from the date of myeloma diagnosis to the date of disease progression or death from any cause, censoring at the time last known to be alive and progression-free. Group differences in survival were assessed with log-rank tests, and median follow-up time was calculated according to the reverse Kaplan–Meier method. Survival curves were estimated using the Kaplan–Meier method, with variance and confidence intervals (CI) estimated using Greenwood formula. Cox regression was used for time-to-event outcomes; HRs and 95% CI were reported with Wald *P* values for covariates. The Benjamini–Hochberg correction was used to adjust for multiple hypothesis testing. Wilcoxon rank-sum and Fisher exact tests were used for CH association with continuous and categorical variables, respectively. Ordinal variables with three or more groups were tested for association with CH using a Kruskal–Wallis test for singly ordered contingency tables. *P* values were two sided, and those <0.05 were considered statistically significant. All data were analyzed using R version 3.5.0 (R Core Team).

### Data Availability

Genomic data of patients with multiple myeloma enrolled within the CoMMpass trial (NCT01454297) were generated as part of the MMRF Personalized Medicine Initiative (https://research.themmrf.org). The entire computational analysis was performed and documented in a Jupyter notebook. It has been made publicly available: https://github.com/getzlab/MMRF_CHIP.

## Results

### Baseline Characteristics of the MMRF Cohort

The MMRF cohort consisted of 986 patients with newly diagnosed multiple myeloma with both BM and PB DNA available for analysis. The median age was 63 years (range: 27–93), the ratio of females to males was 2:3, and median follow-up was 4.99 years (range: 0.14–8.07 years; Table 1). Five hundred and twenty-nine patients underwent ASCT with a median follow-up of 5.5 years (range: 0.35–7.98), and 457 patients did not receive a transplant with a median follow-up of 4.96 years (range: 0.14–8.07). Two patients received an allogeneic stem cell transplant and were thus excluded from the survival analyses. The median OS of the whole cohort was not reached while the median PFS was 3.1 years. In transplanted patients, the median OS was not reached [95% CI, not reached (NR)–NR] and the median PFS was 4.2 years (95% CI: 3.9–4.7), whereas in the non-transplanted patients, the median OS was 4.6 years (95% CI: 4.1–5.4) and median PFS was 1.9 years (95% CI: 1.7–2.2).

**TABLE 1:** Baseline patient demographics

		CH		
	Total *n* = 986 (%)	No CH *n* = 887 (90)	CH *n* = 99 (10)	*P value*
Age				
Median (range)	63 (27–93)	62 (27–93)	69 (38–88)	*<0.001* ^a^
Gender				
Female	390 (40)	357 (40)	33 (33)	*0.19* ^b^
Male	596 (60)	530 (60)	66 (67)	
Race				
African American	136 (14)	122 (14)	14 (14)	*0.28* ^b^
American/Alaskan Native	2 (0)	1 (0)	1 (1)	
Asian	15 (2)	13 (1)	2 (2)	
Other	42 (4)	40 (5)	2 (2)	
White	621 (63)	553 (62)	68 (69)	
*Missing*	*170 (17)*	*158 (18)*	*12 (12)*	
ECOG performance status				
0	253 (26)	232 (26)	21 (21)	*0.093* ^c^
1	347 (35)	308 (35)	39 (39)	
2	84 (9)	75 (8)	9 (9)	
3	34 (3)	28 (3)	6 (6)	
4	6 (1)	5 (1)	1 (1)	
*Missing*	*262 (27)*	*239 (27)*	*23 (23)*	
IMWG risk				
0	350 (35)	312 (35)	38 (38)	*>0.99* ^c^
1	197 (20)	179 (20)	18 (18)	
2	111 (11)	98 (11)	13 (13)	
*Missing*	*328 (33)*	*298 (34)*	*30 (30)*	
R-ISS stage				
1	170 (17)	158 (18)	12 (12)	*0.42* ^c^
2	445 (45)	402 (45)	43 (43)	
3	75 (8)	68 (8)	7 (7)	
*Missing*	*296 (30)*	*259 (29)*	*37 (37)*	
High-risk cytogenetics				
Yes	279 (28)	250 (28)	29 (29)	*0.81* ^a^
No	707 (72)	637 (72)	70 (71)	
Beta-2 microglobulin				
Median (range)	3.400 (0.100–37.900)	3.403 (0.100–37.900)	3.400 (0.230–29.100)	*>0.99* ^a^
*Missing*	*34 (3)*	*26 (3)*	*8 (8)*	
LDH				
Median (range)	2.9 (0.2–32.1)	2.9 (0.2–32.1)	2.7 (1.2–10.0)	0.056^a^
*Missing*	*154 (16)*	*133 (15)*	*21 (21)*	
Recurrent bacterial infections				
Yes	20 (2)	14 (2)	6 (6)	*0.011* ^b^
No	966 (98)	873 (98)	93 (94)	
Group 1^d^				
Yes	92 (9)	74 (8)	18 (18)	*0.006* ^b^
No	894 (91)	811 (92)	83 (82)	
Group 2^d^				
Yes	25 (3)	22 (2)	3 (3)	*0.74* ^b^
No	961 (97)	863 (98)	98 (97)	
Group 3^d^				
Yes	56 (6)	52 (6)	4 (4)	*0.65* ^b^
No	930 (94)	833 (94)	97 (96)	
Group 1, 2, or 3^d^				
Yes	160 (16)	138 (16)	22 (22)	*0.12* ^b^
No	826 (84)	747 (84)	79 (78)	

Abbreviations: IMWG, International Myeloma Working Group; R-ISS, Revised International Staging System.

^a^Wilcoxon rank-sum test.

^b^Fisher exact test.

^c^Cochran–Armitage test.

^d^Group 1: ischemic heart disease, coronary artery disease, myocardial infarction, chest pain—cardiac, nstemi, acute coronary syndrome (coronary artery bypass graft), acute coronary syndrome, coronary artery disease chest pain, acute myocardial infarction, cardiac ischemia; Group 2: cerebrovascular disease, transient ischemic attack, cerebrovascular accident, acute cerebrovascular accident, acute ischemic stroke, ischaemic stroke; Group 3: deep vein thrombosis/thromboembolism, deep vein thrombosis, pulmonary embolus, grade 3 thromboembolic event (right femoral dvt), thromboembolic event, thromboembolic event (unspecified).

### Identification of CH in Patients with Newly Diagnosed Multiple Myeloma

**FIGURE 1:**
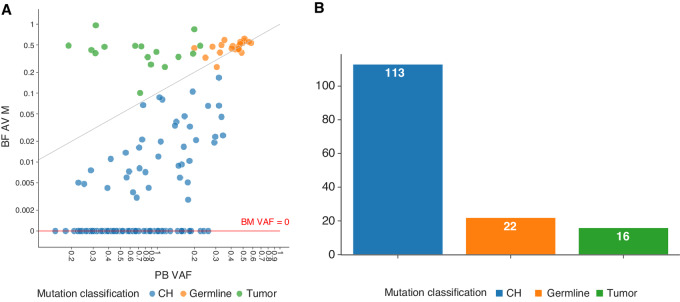
Classification of mutation origins. **A,** Scatterplot of observed VAFs in the PB and BM samples (*n* = 151). Colors specify the classification of each mutation by the winning model: CH (blue), germline (orange), or tumor (green). The scatterplot is in the log scale, with a small offset artificially labeled at *y* = 0 to visualize VAFs of 0 in select BM samples. **B,** Bar graph depicting total number of mutations classified as either CH, germline, or tumor.

**FIGURE 2:**
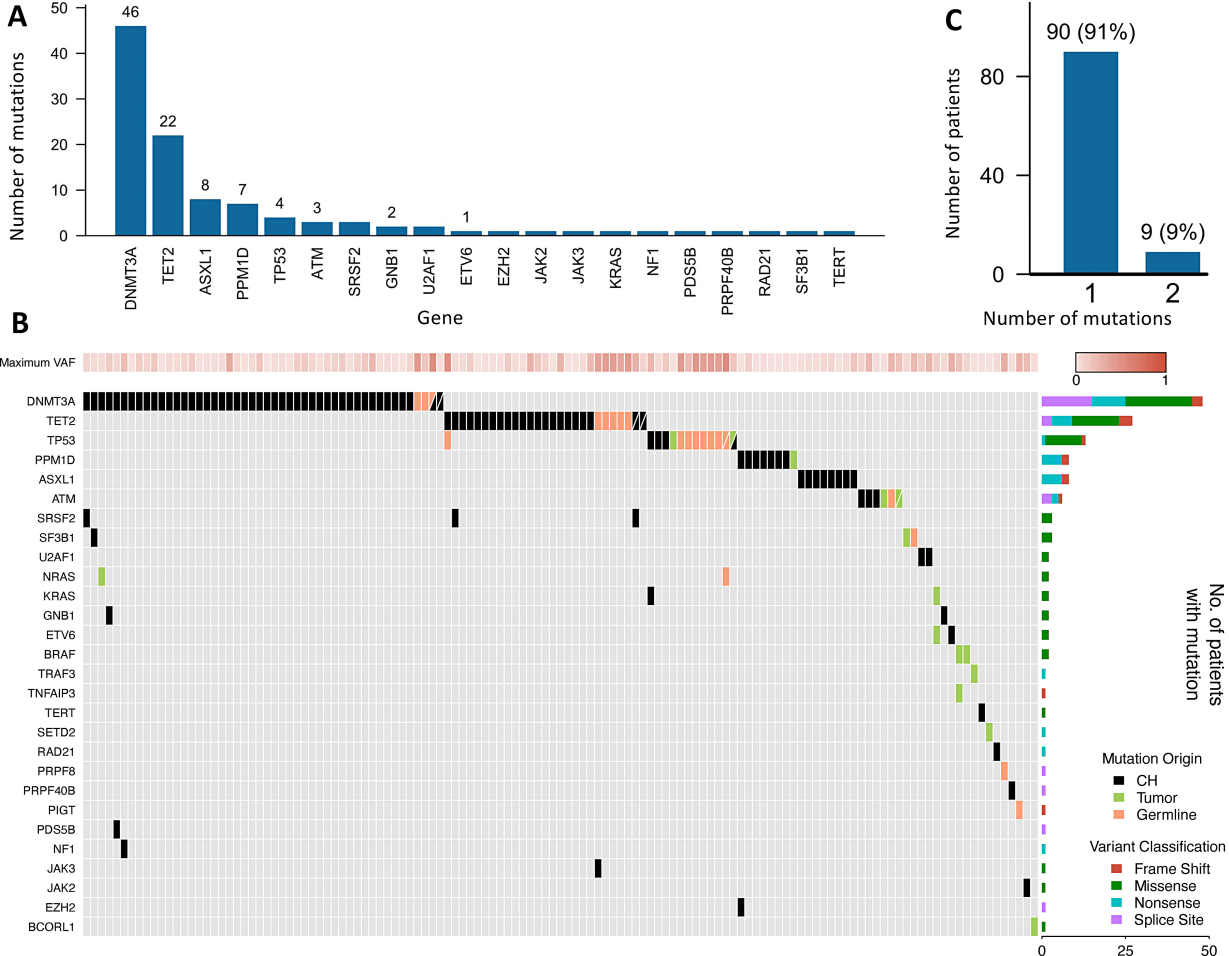
The mutational spectrum of CH in 986 patients with multiple myeloma. **A,** The total number of patients harboring one or more mutations in each gene. **B,** Number of patients harboring mutations in one or two different genes. **C,** Commutation plot showing CH, tumor and germline mutations present in 129 patients: each column represents a single patient. The top row denotes the maximum VAF in each patient, with darker shades of red indicating higher VAF. The bar graph on the right designates the percentage of the different mutation subtypes for each gene out of all detected mutations.

To accurately identify somatic mutations not originating in tumor cells, we developed a Bayesian method that can probabilistically classify mutations to either germline, somatic in the cancer cells, or somatic in the CH clone. We started with the general expectation that (i) germline mutations should have a 50% allele fraction in both BM and PB samples; (ii) BM tumor mutations should have a greater allele fraction in the BM than the PB sample; and (iii) CH mutations should have a greater allele fraction in the PB than the BM sample. This method took into account tumor purity, TiN contamination, and local CNV to create a likelihood model to accurately distinguish CH mutations from the other mutation sources. Using this method, we analyzed the identified 145 candidate CH mutations and predicted 111 of them as being CH, 17 tumor, and 17 germline (Fig. 1A and B). Among all 986 patients, 99 patients (∼10%) harbored the 111 CH mutations (Supplementary Table S3). In the transplant cohort, CH was detected in 40/529 (7.56%) patients, compared with 59/457 (12.91%) in non-transplant patients. Consistent with prior reports, the most commonly mutated genes were *DNMT3A*, *TET2*, *ASXL1*, *PPM1D,* and *TP53*, together constituting 81% (90/111) of the identified CH mutations (Fig. 2A and B). Most patients had only a single CH mutation, and 9 patients had two CH mutations(Fig. 2C).

**FIGURE 3:**
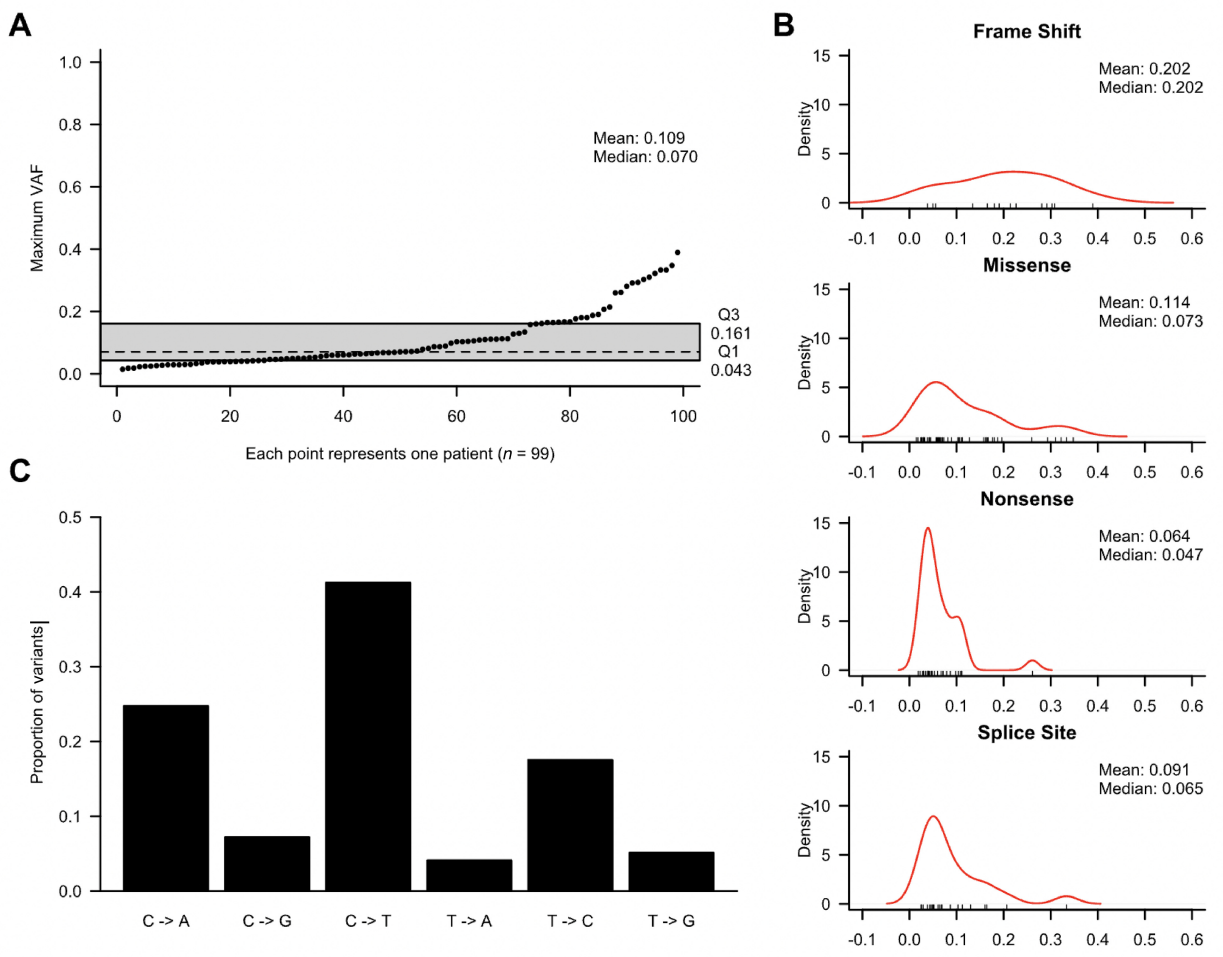
Mutational properties. **A,** The maximum VAF attained by each of the 99 patients with CH. **B,** Distribution of VAF among different variants. **C,** Distribution of the types of single-nucleotide bp changes seen in all detected mutations.

Given that WES provides lower average coverage (113X) compared with targeted sequencing (978X in our prior publication; ref. 25), it is likely that a number of mutations in the 2%–5% VAF range or lower are not reliably detectable in this cohort, likely explaining the difference in CH frequency between the two reports. The identified variants had a median VAF of 7% and mean of 10.9% (Fig. 3A). There were 97 SNVs (87% of all mutations), divided into 47 missense (median VAF: 7.3%), 29 nonsense (median VAF: 4.7%), and 21 splice-site (median VAF: 6.5%) mutations (Fig. 3B). The remaining 14 mutations were frameshift indels (median VAF: 20.2%), including 10 deletions and 4 insertions. Around 40% of mutations were C>T substitutions consistent with prior observations that this is the dominant mutagenic signature in HSCs (Fig. 3C). The overall type and distribution of CH mutations were consistent with previous reports (25).

### Clinical Associations with CH in Newly Diagnosed Myeloma

The presence of CH was associated with older age, with a median age of 69 years in those with CH compared with 62 years in those without (Wilcoxon rank-sum test, *P* < 0.001). As expected, the median age of patients who received an ASCT was lower than those who did not (60 vs. 67 years), which likely explains the slightly higher prevalence of CH (12.91%) detected in the non-transplanted group. Across the entire cohort, univariate analysis showed that patients with CH had an increased risk of recurrent bacterial infections (*P* = 0.011) and cardiovascular disease (*P* = 0.0031) but not with cerebrovascular disease (*P* = 0.73) or coagulopathies (*P* = 0.65). Finally, CH present at diagnosis was not associated with known risk factors for myeloma progression, including beta-2 microglobulin, lactate dehydrogenase (LDH), International Staging System (ISS) score, and high-risk cytogenetic abnormalities (Table 1).

**FIGURE 4:**
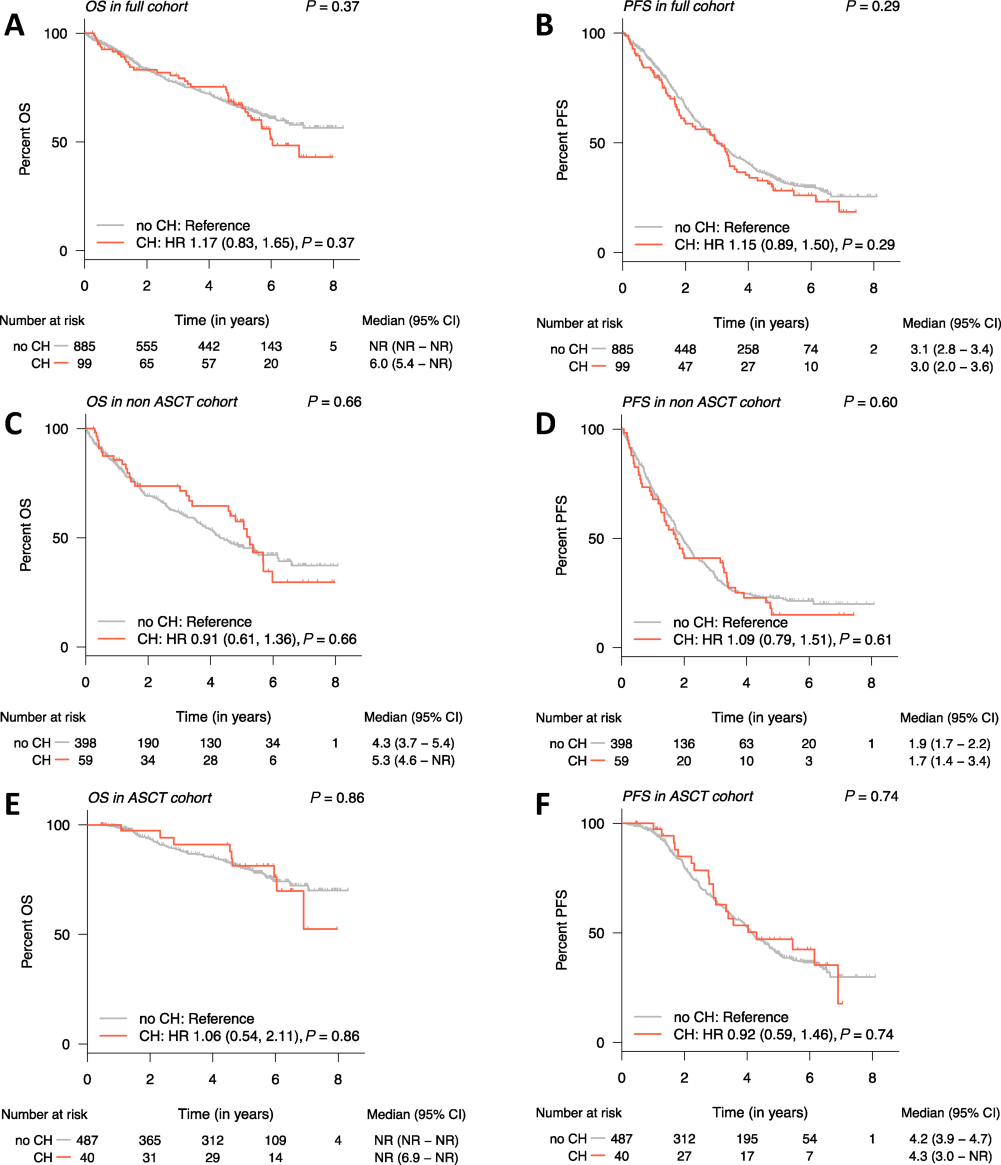
OS and PFS of non-transplanted and transplanted patients with multiple myeloma with respect to CH and IMiD treatment. OS (**A**) and PFS (**B**) of all patients with CH versus those who do not have CH. OS (**C**) and PFS (**D**) of non-transplanted patients with CH versus those who do not have CH. OS (**E**) and PFS (**F**) among transplanted patients with CH versus those who do not have CH.

Across the full cohort, we did not see a significant association between CH and OS (median OS of 6 years in those with CH vs. not reached in those without CH, *P* = 0.37; Fig. 4A) or PFS (median PFS 3 and 3.1 years for those with and without CH, respectively, *P* = 0.29; Fig. 4A). Among the 457 non-transplanted patients, the presence of CH was not significantly associated with OS (median OS 5.3 years with CH vs. 4.3 years without CH, *P* = 0.66; Fig. 4C) or PFS (median PFS 1.7 years with patients with CH vs. 1.9 years without CH, *P* = 0.60; Fig. 4D). Similarly, among the 527 transplanted patients, the median OS was not reached for patients with and without CH (*P* = 0.86; Fig. 4E). PFS was also not significantly different among transplanted patients (median PFS 4.3 years with CH vs. 4.2 years in those without CH, *P* = 0.74; Fig. 4F). Furthermore, stratifying by clone size (i.e., VAF), we still did not observe an impact of clone size on either OS or PFS. When looking at patients with mutations in the most common genes *DNMT3A*, *TET2,* and *ASXL1*, we did not find a significant association with PFS or OS, which is likely related to our small numbers and short follow-up time.

We have previously reported that presence of CH is associated with shorter PFS and OS after ASCT, but that effect is ameliorated in patients who received IMiD maintenance (25). Therefore, we examined the interaction between CH and IMiD exposure in patients with both transplanted and non-transplanted multiple myeloma. The use of IMiD for induction in non-transplanted patients or as maintenance post-ASCT was associated with improved PFS and OS, irrespective of whether CH was present (Supplementary Fig. S3 and S4).

### CH and Risk of Secondary Malignancy

Out of 986 patients, 8 patients developed a second primary hematologic malignancy: 3 AML, 3 MDS, 1 chronic myeloid leukemia, and 1 who initially developed MDS then developed diffuse large B-cell lymphoma (Supplementary Table S4). Furthermore, 25 patients developed a secondary solid malignancy at some point, including 10 skin cancers, 2 breast cancer, 2 colon cancer, 2 prostate cancer, 2 lung cancer, and 1 each of hepatocellular, bladder, esophageal, thyroid, pancreatic, oropharyngeal and granular cell tumor. CH at multiple myeloma diagnosis was not associated with increased risk of developing a new hematologic malignancy (*P* = 0.57) or a new solid malignancy (*P* = 0.73). Only one AML case had a CH mutation at the time of multiple myeloma diagnosis, and the rest were CH-free. Samples collected at the time of secondary hematologic malignancy diagnosis were not available for analysis to allow comparison of the malignant clone with prior CH.

### Evolution of CH During Treatment

To evaluate the clonal evolution of CH during the course of multiple myeloma–directed treatment, we analyzed serial samples in 52 patients (36 transplant and 16 non-transplant patients; Supplementary Table S5). Forty-eight patients had PB samples collected at two timepoints, and 4 patients had samples at three or more timepoints. The median time between the first and second sample was 3.12 years (range: 0.97–5.43 years). Among patients with multiple samples, only 3/52 (5.76%) patients had CH at the time of multiple myeloma diagnosis (median age of the 52 patients was 63 years). Following the initiation of therapy, CH mutations were present in 10 additional patients (total of 13/52, 25%) with a median age of 67 years for all 52 patients (Supplementary Table S6). This prevalence of CH in patients having received anti-multiple myeloma therapy is significantly higher than the approximately 6%, which is expected in the general population of the same age group (60–69 years; ref. 16; *P* = 0.013; Fisher exact two-sided test). The 13 patients with CH received a variety of intervening therapies: 8 patients underwent transplant, and 12 received IMiD therapy at some point (Supplementary Fig. S5). The most common emergent mutation was in *DNMT3A*, found in five of the 10 new CH cases, suggesting that standard multiple myeloma therapy does not promote CH mutations different from those seen in the general population. While most patients had expanding or newly emergent clones, one patient (patient MMRF_1079) had a *DNMT3A*-mutant clone that shrank while on lenalidomide and carfilzomib, starting at a VAF of 16% and significantly dropping to 5% (*P* = 0.0017; Fisher exact two-tailed test) and 4% (*P* = 0.00025; Fisher exact two-tailed test) at subsequent timepoints (Supplementary Tables S5 and S6).

## Discussion

In this study, we report a novel algorithm for detecting CH mutations using data from PB and tumor sequencing. By leveraging our algorithm on WES of PB and BM samples of a large cohort of patients with newly diagnosed multiple myeloma, we were able to remove biological noise due to germline variants and circulating tumor cells that may contaminate PB samples, which has not been done in prior CH studies. In this large, multicenter, cohort of patients with newly diagnosed multiple myeloma, we detected CH at a prevalence of 10% with a median age of 63 years. The difference in prevalence seen in this cohort compared with other published datasets can likely be explained by variations in median age as well as differences in sequencing depth, number of genes assayed, and mutation detection sensitivity (Supplementary Fig. S6). Overall, the distribution of CH mutations was similar to that seen in otherwise healthy individuals of a similar age and distinct from that seen in chemo-exposed patients (43, 44). We also identified associations between presence of CH and risk of cardiovascular disease as well as recurrent infections, consistent with prior reports, suggesting that the presence of CH may be associated with increased non-multiple myeloma morbidity (22, 45–47).

Our prior work, utilizing a cohort from a single institution, found that having CH at the time of ASCT was associated with a shorter PFS and OS in patients with multiple myeloma (25). In this study, we were interested in examining this association in a larger number of patients from multiple centers, investigating the effect of CH on the long-term outcomes of patients including those who did not undergo ASCT, and understanding the temporal changes in CH during multiple myeloma–directed therapy. There are several important differences between our prior work and the current study. In our prior study, the follow-up time was around 10 years, compared with around 5 years in this study, which may have not provided enough time to see a significant difference in PFS and OS. Moreover, the prior study only included transplanted patients who received ASCT between 2003 and 2011 while this study included both transplanted and non-transplanted patients diagnosed with multiple myeloma in 2011 and beyond, which reflects a very different treatment era for myeloma with much better outcomes. This includes incorporation of anti-CD38 mAbs in the induction regimen and maintenance in some cases, as well as consistently incorporating IMiDs in induction and maintenance, which reliably improve outcomes for everyone (48–52). In addition, we checked for CH in the previous study from the stem cell products mobilized right before ASCT, after patients were exposed to 1–3+ lines of therapy, compared with this study which looked for CH in the PB at time of diagnosis in patients with treatment-naïve multiple myeloma. This could have led to the higher incidence of CH and even worse outcomes seen in our prior study. Moreover, we did not have tumor sample available for sequencing in the prior study, and thus it is possible that patients with aggressive multiple myeloma could have had tumor cells present at the time of stem cell collection with mutations that were erroneously called CH. The presence of active myeloma and circulating plasma cells at the time of transplant has previously been associated with inferior OS and PFS (53). There are also differences in the sequencing platforms used that could explain the divergent results. The WES used in this study, as compared with targeted sequencing in our prior work, led to an average coverage that was approximately 10-fold lower and thus a lower detection rate of CH clones, particularly those with a VAF less than 5%. Therefore, it is possible that we did not detect an effect on OS or PFS in part due to lower sequencing coverage leading to some patients being falsely considered CH-negative when in fact they did harbor smaller CH clones that were below our detection limit. In further support of this, in our prior report, we were only able to detect an association between CH and disease progression and survival when smaller clones (VAF of 1%–2%) were included in the analyses, made possible through deep targeted sequencing. Of note, the prevalence of CH in our prior report would decrease from around 22% to 14% if we were to only include the clones of VAF ≥2%, which is closer to the 10% detected by WES in this study. Whether these methodologic differences explain the previously observed survival associations, and the biological explanation for how small clones might induce outsized clinical effects, will require additional investigation.

Importantly, this study confirms prior findings of a significant clinical benefit to IMiD therapy (54, 55). This benefit was seen irrespective of CH status, which is in agreement with our prior report. While emerging data have suggested that IMiD therapy may promote selection and clonal evolution of secondary myeloid neoplasms in specific cases, namely in patients with *TP53*-mutant CH (56), our data would suggest that most patients with CH benefit from IMiD-based therapy. Thus, the current weight of evidence would suggest that the presence of any CH should not preclude patients with multiple myeloma from receiving this beneficial therapy.

The dynamic evolution of CH in healthy individuals has been described recently (57, 58) but CH evolution in patients with multiple myeloma undergoing therapy has not been well studied. Consistent with prior reports that chemo-exposed patients have higher rates of CH, we saw expansion or emergence of mutant clones in most patients. However, unlike reports in patients heavily exposed to cytotoxic agents, we did not see a predominance of *TP53* and *PPM1D* mutations expand, likely reflecting the unique plasma cell–directed mechanism of action of most agents employed in the treatment of multiple myeloma (18, 44). Multiple myeloma therapy, which primarily included IMiDs, proteasome inhibitors, and ASCT, led to a significant increase in CH prevalence that was mainly driven by *DNMT3A* clonal expansion. Prior work has shown that patients with multiple myeloma who go on to develop secondary leukemias may already carry stem cell mutations at very low VAF years prior to developing the secondary malignancy (25, 59). Six of 7 patients with a *DNMT3A* mutation identified at the second timepoint had undergone ASCT, which is consistent with reports of ASCT allowing *DNMT3A* clones to grow (60). Whether multiple myeloma therapy led to the development of new mutations or the selection of preexistent therapy-resistant clones that were below the detection threshold is unknown. It has also been suggested that *DNMT3A*-mutant clones may have an engraftment and growth advantage in the immediate post-ASCT setting (61). The role that clonal evolution of CH during multiple myeloma treatment plays in the risk of developing secondary leukemias and other clinical outcomes remains to be elucidated.

In summary, we detected CH in approximately 10% of patients with treatment-naïve newly diagnosed multiple myeloma using a novel computational algorithm to confidently assign candidate CH mutations to CH, tumor, or germline. The presence of CH was not associated with inferior OS or PFS in either transplanted or non-transplanted patients, and all patients benefited from IMiD-based therapies, irrespective of CH status. The negative clinical impact of CH seems to be mitigated in light of significant advances in myeloma therapies. Yet, throughout the course of treatment, patients with multiple myeloma acquire and/or evolve previously undetected CH clones, particularly those with *DNMT3A* mutations, indicating that multiple myeloma treatment may accelerate the natural course of CH, the clinical significance of which will require further work.

## Supplementary Material

Supplementary Methods 1 Supplementary Methods

Supplementary Figure 1 Depiction of cell mixtures for the normal (PB) sample and tumor (BM) sample showing effect of tumor purity and tumor-in-normal (TiN) contamination. Relative areas represent DNA fractions of different cell types.

Supplementary Figure 2 Depiction of cell mixtures for PB and BM samples.

Supplementary Figure 3 Effect of IMiDs and CH on progression-free survival.

Supplementary Figure 4 Effect of IMiDs and CH on overall survival.

Supplementary Figure 5 Temporal changes in CH in MM.

Supplementary Figure 6 Power to detect CH mutations.

Supplementary Table 1 List of queried genes and variants to determine the presence of CH.

Supplementary Table 2 List of genes in the targeted sequencing panel.

Supplementary Table 3 List of 145 candidate mutations in the baseline samples classified as either CH, Tumor or Germline.

Supplementary Table 4 Characteristics of the 8 patients who developed a second primary malignancy.

Supplementary Table 5 List of mutations classified as CH in the sequential samples.

Supplementary Table 6 Temporal changes in CH clones in 13 patients with sequential samples.
