# Supplementary material for: Clinical Outcomes and Evolution of Clonal Hematopoiesis in Patients with Newly Diagnosed Multiple Myeloma
Source: Cancer Res Commun. 2023 Dec 18;3(12):2560–71. doi: 10.1158/2767-9764.CRC-23-0093 (PMC10730502; doi:10.1158/2767-9764.CRC-23-0093)
Supplement: Supplementary Methods 1 — Supplementary Methods [file crc-23-0093-s01.docx]

# SUPPLEMENTARY METHODS

## Computational analysis

The RealignmentFilter tool is based on the BLAT filter^1^.

## Artifact analysis

We removed technical artifacts from our candidate CH mutations using two methods: a panel of normals (PoN)^2,3^ and the Getz lab’s BLAT^1^ filter tool. We constructed a PoN using patients in the MMRF cohort who did not have any detectable CH and were under age 50. Artifacts captured by both methods were removed from the final list of true CH mutations without further review. Any artifact captured by a single filtering method was reviewed in IGV^4^ for evidence of mapping to other regions in the genome. If no such evidence was found in IGV, the mutation was retained as true CH.

## Likelihood model

All candidate CH mutations have variant alleles in the blood. Some candidate CH mutations have no variant alleles at the same loci in matched BM samples. Within a subset of serial PB samples we found the absence of genetic variants in early biopsies followed by CH mutations in later biopsies. The absence of variant alleles is not definitive because there is greater uncertainty in zero- and low-read counts. Because of this uncertainty, we added a pseudocount of 1 to all alternate and reference allele counts to avoid expected VAF values of 0 and smooth our likelihood functions. Modified expected VAFs were calculated as follows: [
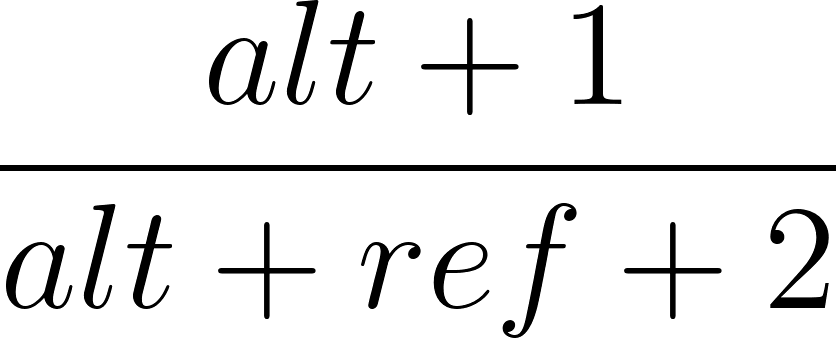
](https://www.codecogs.com/eqnedit.php?latex=%5Cfrac%7Balt%2B1%7D%7Balt%2Bref%2B2%7D#0). VAF is Beta-distributed, Beta(VAF, *a*, *b*), and no changes were made to the Beta distribution’s original shape parameters [
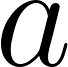
](https://www.codecogs.com/eqnedit.php?latex=a#0) and [
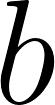
](https://www.codecogs.com/eqnedit.php?latex=b#0).

## Two-way contamination: deriving alpha* and beta*

DeTiN^5^ was designed to measure tumor-in-normal (TiN) contamination. TiN is defined as a relative fraction of tumor DNA in the PB sample to tumor DNA in the BM sample:

[
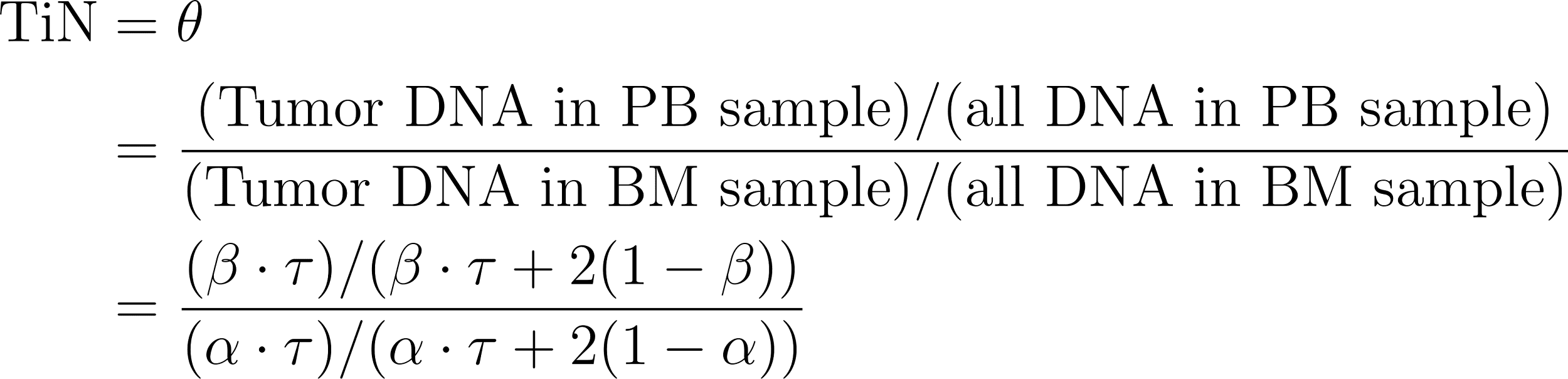
](https://www.codecogs.com/eqnedit.php?latex=%5Cbegin%7Balign*%7D%20%5Ctext%7BTiN%7D%20%26%3D%20%5Ctheta%20%5C%5C%5C%5C%20%26%3D%20%5Cfrac%7B(%5Ctext%7BTumor%20DNA%20in%20PB%20sample%7D)%2F(%5Ctext%7Ball%20DNA%20in%20PB%20sample%7D)%7D%7B(%5Ctext%7BTumor%20DNA%20in%20BM%20sample%7D)%2F(%5Ctext%7Ball%20DNA%20in%20BM%20sample%7D)%7D%20%5C%5C%5C%5C%20%26%3D%20%5Cfrac%7B(%5Cbeta%20%5Ccdot%20%5Ctau)%2F(%5Cbeta%20%5Ccdot%20%5Ctau%20%2B%202(1-%5Cbeta)%20)%20%7D%20%7B(%5Calpha%20%5Ccdot%20%5Ctau)%2F(%5Calpha%20%5Ccdot%20%5Ctau%20%2B%202(1-%5Calpha)%20)%20%7D%20%5Cend%7Balign*%7D%20#0)

[
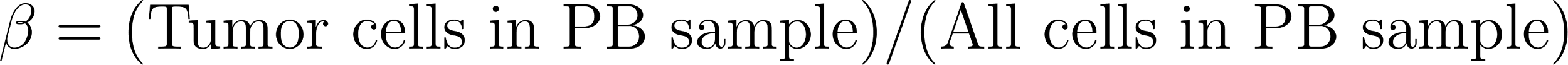
](https://www.codecogs.com/eqnedit.php?latex=%20%5Cbeta%20%3D%20(%5Ctext%7BTumor%20cells%20in%20PB%20sample%7D)%2F(%5Ctext%7BAll%20cells%20in%20PB%20sample%7D)%20#0)

[
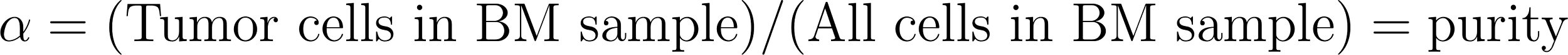
](https://www.codecogs.com/eqnedit.php?latex=%5Calpha%20%3D%20(%5Ctext%7BTumor%20cells%20in%20BM%20sample%7D)%2F(%5Ctext%7BAll%20cells%20in%20BM%20sample%7D)%20%3D%20%5Ctext%7Bpurity%7D#0)

[
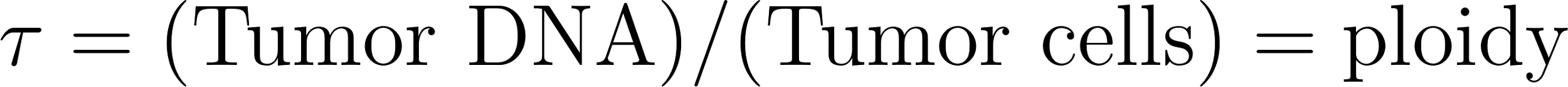
](https://www.codecogs.com/eqnedit.php?latex=%20%5Ctau%20%3D%20(%5Ctext%7BTumor%20DNA%7D)%2F(%5Ctext%7BTumor%20cells%7D)%20%20%3D%20%5Ctext%7Bploidy%7D#0)

The two values that provide information on sample contamination are [
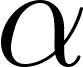
](https://www.codecogs.com/eqnedit.php?latex=%5Calpha#0) and [
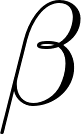
](https://www.codecogs.com/eqnedit.php?latex=%5Cbeta#0). We solve for [
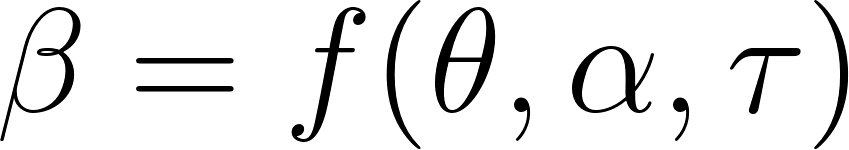
](https://www.codecogs.com/eqnedit.php?latex=%5Cbeta%3Df(%5Ctheta%2C%5Calpha%2C%5Ctau)#0), the fraction of tumor cells in the PB sample:

[
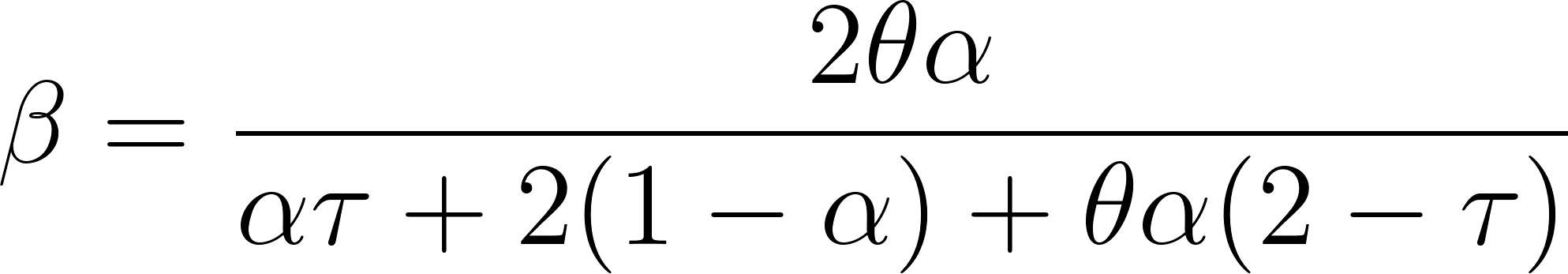
](https://www.codecogs.com/eqnedit.php?latex=%20%5Cbeta%20%3D%20%5Cfrac%7B2%20%5Ctheta%20%5Calpha%7D%20%7B%5Calpha%20%5Ctau%20%2B%202(1-%5Calpha)%20%2B%20%5Ctheta%20%5Calpha%20(2-%5Ctau)%20%20%7D%20#0)

This expression for [
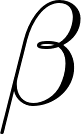
](https://www.codecogs.com/eqnedit.php?latex=%5Cbeta#0) is a bit non-intuitive but has reasonable asymptotic values. We see that as [
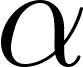
](https://www.codecogs.com/eqnedit.php?latex=%5Calpha#0) decreases, [
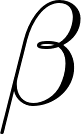
](https://www.codecogs.com/eqnedit.php?latex=%5Cbeta#0) also decreases: If we see fewer tumor cells in a tumor sample, we expect to see fewer tumor cells contaminating the blood sample. Because we assume there is no copy number variation in CH, note that [
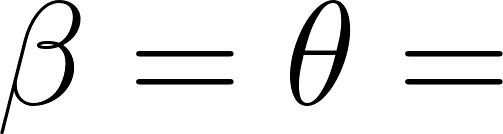
](https://www.codecogs.com/eqnedit.php?latex=%5Cbeta%3D%5Ctheta%3D#0)TiN when [
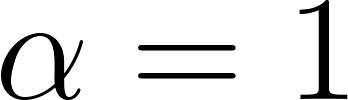
](https://www.codecogs.com/eqnedit.php?latex=%5Calpha%3D1#0) and [
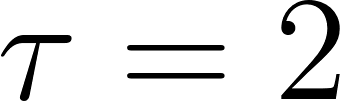
](https://www.codecogs.com/eqnedit.php?latex=%5Ctau%3D2#0).

Because there can be CNVs in the tumor, we characterize the site of each candidate mutation in terms of DNA from normal and tumor cells. We define [
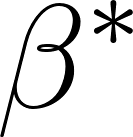
](https://www.codecogs.com/eqnedit.php?latex=%5Cbeta%5E*#0) as the local DNA fraction of tumor cells in the PB sample:

[
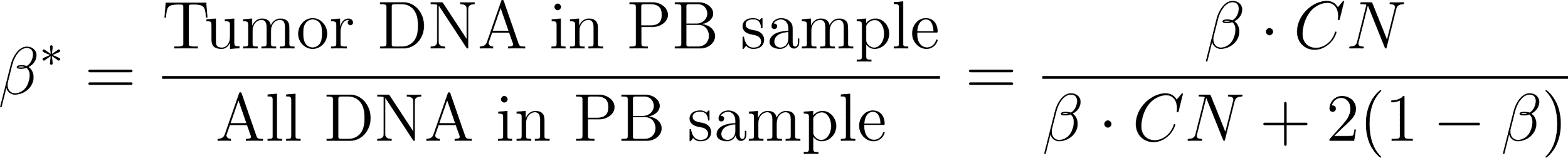
](https://www.codecogs.com/eqnedit.php?latex=%5Cbeta%5E*%20%3D%20%5Cfrac%7B%5Ctext%7BTumor%20DNA%20in%20PB%20sample%7D%7D%7B%5Ctext%7BAll%20DNA%20in%20PB%20sample%7D%7D%20%3D%20%5Cfrac%7B%5Cbeta%20%5Ccdot%20CN%7D%7B%5Cbeta%20%5Ccdot%20CN%20%2B%202(1-%5Cbeta)%7D#0)

Likewise, we define [
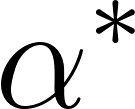
](https://www.codecogs.com/eqnedit.php?latex=%5Calpha%5E%7B*%7D#0) as the local DNA fraction of tumor cells in the tumor sample:

[
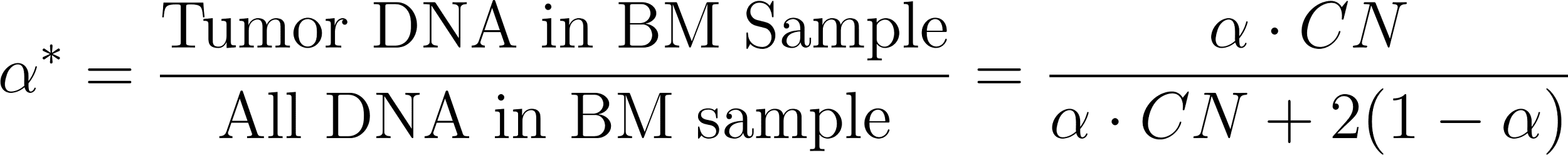
](https://www.codecogs.com/eqnedit.php?latex=%5Calpha%5E*%20%3D%20%5Cfrac%7B%5Ctext%7BTumor%20DNA%20in%20BM%20Sample%7D%7D%7B%5Ctext%7BAll%20DNA%20in%20BM%20sample%7D%7D%20%3D%20%5Cfrac%7B%5Calpha%20%5Ccdot%20CN%7D%7B%5Calpha%20%5Ccdot%20CN%20%2B%202(1-%5Calpha)%7D#0)

Therefore, [
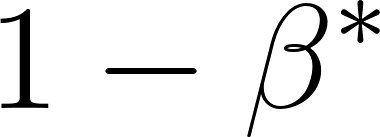
](https://www.codecogs.com/eqnedit.php?latex=1-%5Cbeta%5E%7B*%7D#0) is the fraction of non-tumor DNA in the PB sample and [
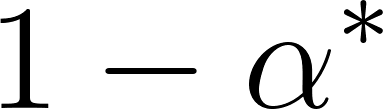
](https://www.codecogs.com/eqnedit.php?latex=1-%5Calpha%5E%7B*%7D#0) is the fraction of non-tumor DNA in the BM sample.

## Uncertain fraction of PB DNA in BM sample: *x*

Although BM samples are first sorted to enrich for myeloma cells, non-tumor cells may still be present in the sorted sample. Since we don’t know what proportion of the remaining non-tumor cells contain PB DNA, we define [
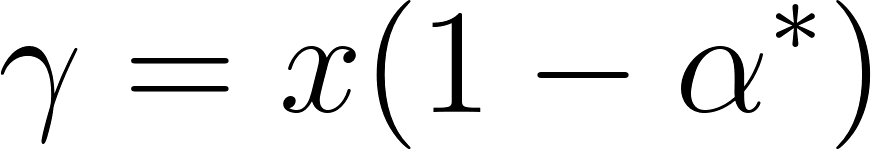
](https://www.codecogs.com/eqnedit.php?latex=%5Cgamma%20%3D%20x(1-%5Calpha%5E%7B*%7D)#0). Under the CH model, [
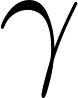
](https://www.codecogs.com/eqnedit.php?latex=%5Cgamma#0) is the fraction of the PB DNA that remains in the BM sample and [
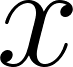
](https://www.codecogs.com/eqnedit.php?latex=x#0) is the contribution of the PB DNA to the non-tumor portion of the BM sample. Since [
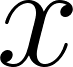
](https://www.codecogs.com/eqnedit.php?latex=x#0) is unknown, we integrate the likelihoods over all possible values. We generate CH likelihoods for values of [
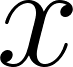
](https://www.codecogs.com/eqnedit.php?latex=x#0) between 0 and 1 and then average these values to calculate the final CH likelihood.

We also considered different a priori expected models of *x* in the likelihood estimates. It is implausible that the contribution of PB to the non-tumor portion of the BM sample is 100%, so we compared several methods for weighing values of *x*. Each model can be described by a probability distribution function *F(x)* on the interval [0,1]. We compared a standard uniform distribution, a piecewise uniform distribution, and a linear probability distribution.

When all contributions of PB DNA between 0% and 100% to the non-tumor portion are given equal weight, we use the standard uniform distribution: [
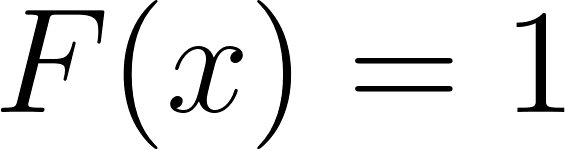
](https://www.codecogs.com/eqnedit.php?latex=F(x)%3D1#0).

A piecewise uniform distribution gives more weight to PB contributions between 0% and 75% and zero weight to PB contributions greater than 75%:

[
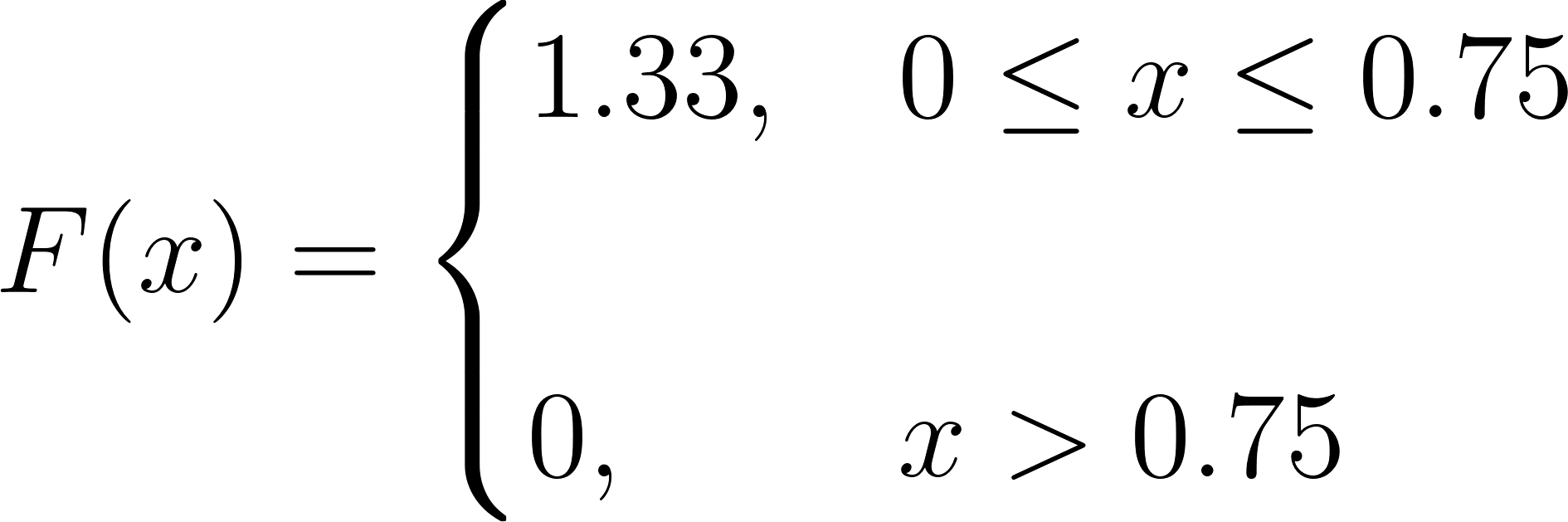
](https://www.codecogs.com/eqnedit.php?latex=F(x)%20%3D%20%5Cbegin%7Bcases%7D%201.33%2C%20%26%200%5Cle%20x%5Cle%200.75%20%5C%5C%5C%5C%5C%5C%200%2C%20%26%20x%20%3E%200.75%5Cend%7Bcases%7D#0)

The linear distribution function, [
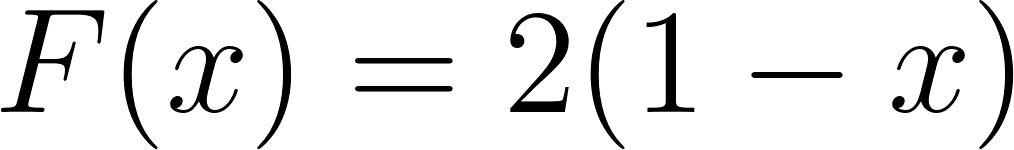
](https://www.codecogs.com/eqnedit.php?latex=F(x)%3D2(1-x)#0), describes an inverse, linear relationship between the PB contribution and its probability. Smaller PB contributions are given more weight than larger PB contributions to the non-tumor portion of BM samples.

In general, our models were robust over different choices of *F(x)*. Mutation classification was consistent, with the exception of two outlying mutations coming from a single patient’s mutations in KRAS. For each correction method, these mutations had lower classifier confidence compared to other mutation classifications: 99.2%, 94.5%, and 84.9%, respectively. These confidence values were consistently the lowest 6 out of 151 mutations. Our review of these KRAS mutations suggests that they are a multiple nucleotide polymorphism (MNP) consisting of p.G12D and p.G13A substitutions. Since MNPs in KRAS are far more common in MM tumors than in CH, we chose for our analysis the weighting method that classified these KRAS mutations as ‘tumor’: the piecewise uniform distribution. The logic here is similar to a posterior prior: a prior that KRAS is from a tumor, which we subsequently used to select the “chopped” *F(x)* as the best model.

## Expected variant allele fraction (VAF)

*Germline model*

We estimated germline het allele fractions in the PB and BM samples. Within tumor DNA a germline het should have allele fraction

[
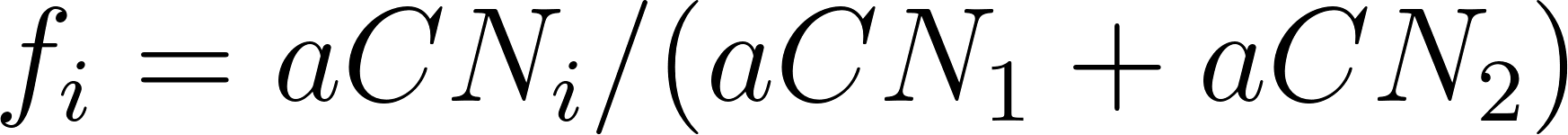
](https://www.codecogs.com/eqnedit.php?latex=f_i%20%3D%20aCN_i%2F(aCN_1%2BaCN_2)#0)

where [
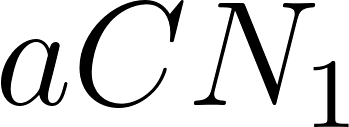
](https://www.codecogs.com/eqnedit.php?latex=aCN_1#0) is the allelic copy number in the tumor for the minor allele, [
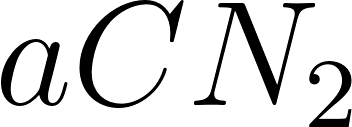
](https://www.codecogs.com/eqnedit.php?latex=aCN_2#0) is the allelic copy number in the tumor for the major allele, and [
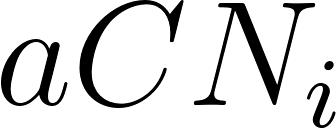
](https://www.codecogs.com/eqnedit.php?latex=aCN_i#0) is the allelic copy number in the tumor of either the minor or major allele. The allelic copy numbers are estimated by ABSOLUTE^6^ for each copy segment.

For the tumor sample, for either the minor or major allele:

[
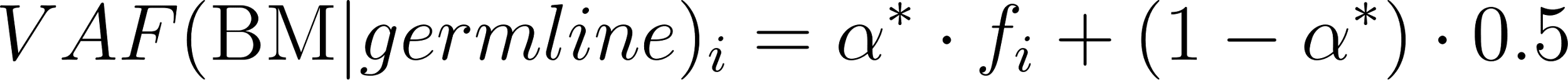
](https://www.codecogs.com/eqnedit.php?latex=VAF(%5Ctext%7BBM%7D%7Cgermline)_i%20%20%3D%20%5Calpha%5E*%20%5Ccdot%20f_i%20%2B%20(1-%5Calpha%5E*)%20%5Ccdot%200.5%20#0).

For the PB sample, for either minor or major allele:

[
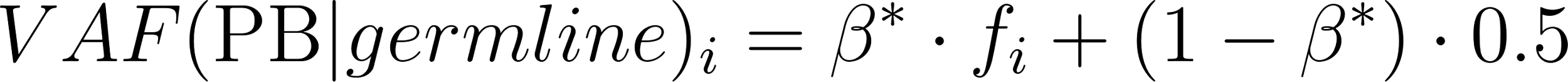
](https://www.codecogs.com/eqnedit.php?latex=VAF(%5Ctext%7BPB%7D%7Cgermline)_i%20%20%3D%20%5Cbeta%5E*%20%5Ccdot%20f_i%20%2B%20(1-%5Cbeta%5E*)%20%5Ccdot%200.5%20#0).

The overall estimate of the germline likelihood model is the maximum of the likelihoods over the minor and major alleles (i.e. we first calculate two likelihood values, one for each [
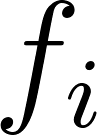
](https://www.codecogs.com/eqnedit.php?latex=f_i#0) and choose [
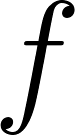
](https://www.codecogs.com/eqnedit.php?latex=f#0) that gives the greater likelihood).

*CH model*

We first define the “true” VAF of CH mutations in PB DNA as [
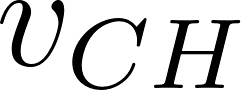
](https://www.codecogs.com/eqnedit.php?latex=v_%7BCH%7D#0), regardless of contamination. The observed VAF of CH mutations is affected by the mixture of PB and BM samples:

[
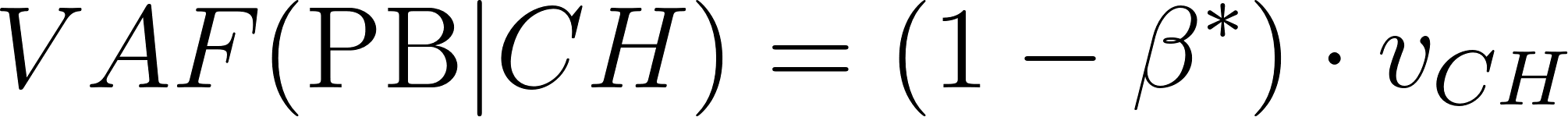
](https://www.codecogs.com/eqnedit.php?latex=%20VAF(%5Ctext%7BPB%7D%7CCH)%20%3D%20(1-%5Cbeta%5E*)%20%5Ccdot%20v_%7BCH%7D%20#0)

The observed VAF of CH mutations in the tumor sample depends on the fraction [
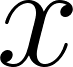
](https://www.codecogs.com/eqnedit.php?latex=x#0) of PB cells in normal cells in the tumor sample:

[
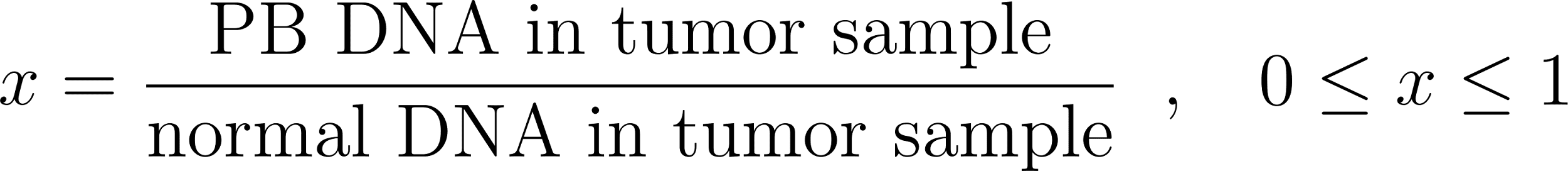
](https://www.codecogs.com/eqnedit.php?latex=%20x%20%3D%20%5Cfrac%7B%5Ctext%7BPB%20DNA%20in%20tumor%20sample%7D%7D%7B%5Ctext%7Bnormal%20DNA%20in%20tumor%20sample%7D%7D%20%5C%3B%5C%3B%2C%20%5C%3B%5C%3B%5C%3B%200%5Cleq%20x%20%5Cleq%201#0)

[
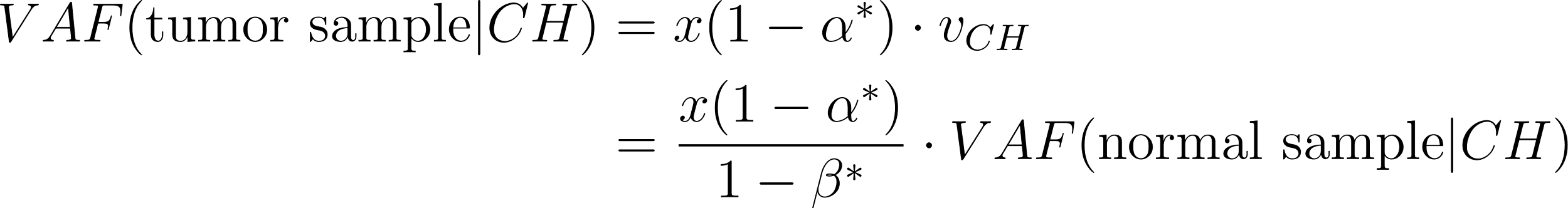
](https://www.codecogs.com/eqnedit.php?latex=%5Cbegin%7Balign*%7D%20VAF(%5Ctext%7Btumor%20sample%7D%7CCH)%20%26%3D%20x(1-%5Calpha%5E*)%20%5Ccdot%20v_%7BCH%7D%20%5C%5C%20%26%3D%20%20%5Cfrac%7Bx(1-%5Calpha%5E*)%7D%7B1-%5Cbeta%5E*%7D%20%5Ccdot%20VAF(%5Ctext%7Bnormal%20sample%7D%7CCH)%20%5Cend%7Balign*%7D%20#0)

*Somatic tumor model*

We also define the “true” VAF of tumor mutations in BM DNA as [
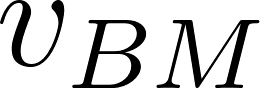
](https://www.codecogs.com/eqnedit.php?latex=v_%7BBM%7D#0), regardless of contamination. The observed VAF of BM mutations in tumor sample also depends on the mixture of PB and BM samples:

[
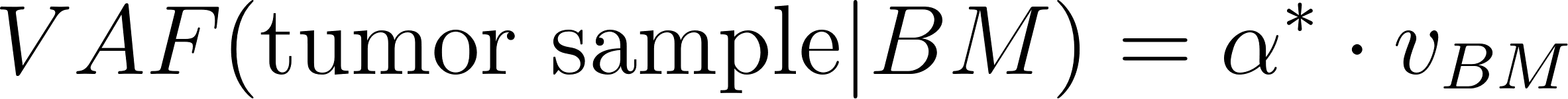
](https://www.codecogs.com/eqnedit.php?latex=%20VAF(%5Ctext%7Btumor%20sample%7D%7CBM)%20%3D%20%5Calpha%5E*%20%5Ccdot%20v_%7BBM%7D%20#0)

[
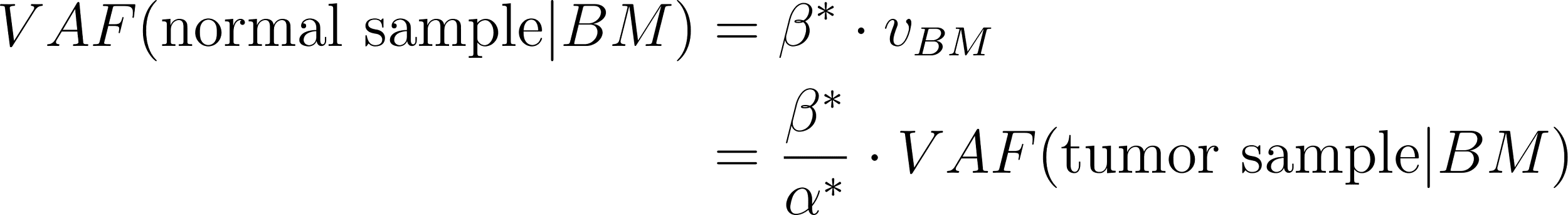
](https://www.codecogs.com/eqnedit.php?latex=%5Cbegin%7Balign*%7D%20VAF(%5Ctext%7Bnormal%20sample%7D%7CBM)%20%26%3D%20%5Cbeta%5E*%20%5Ccdot%20v_%7BBM%7D%20%5C%5C%5C%5C%20%26%3D%20%5Cfrac%7B%5Cbeta%5E*%7D%7B%5Calpha%5E*%7D%20%5Ccdot%20VAF(%5Ctext%7Btumor%20sample%7D%7CBM)%20%5Cend%7Balign*%7D#0)

## Confidence

For each mutation we test three hypotheses and generate three likelihood values. Similarly, for each mutation, we calculate three probabilities based on the likelihood values generated for each hypothesis. The largest probability value corresponds to the large likelihood value and determines the classifier confidence, which we define as the probability of a mutation originating from the most likely source:

[
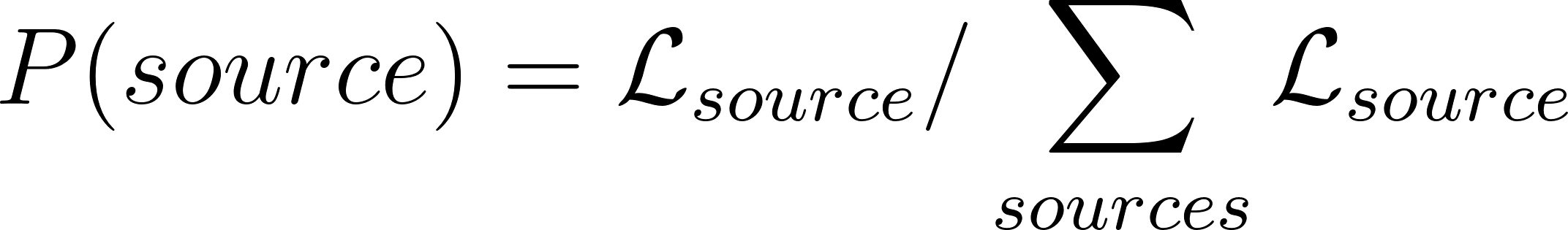
](https://www.codecogs.com/eqnedit.php?latex=P(source)%20%3D%20%5Cmathcal%7BL%7D_%7Bsource%7D%2F%20%5Csum_%7Bsources%7D%5Cmathcal%7BL%7D_%7Bsource%7D#0)

[
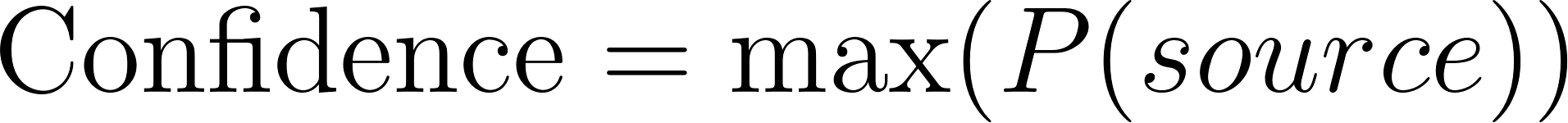
](https://www.codecogs.com/eqnedit.php?latex=%5Coperatorname%7BConfidence%7D%20%3D%20%5Coperatorname%7Bmax%7D(P(source))#0)

## Germline mutations

After detecting candidate somatic CH mutations in PB samples, 19 were classified by our Bayesian model as having a germline origin. However, a subset of mutations (6/19) have VAFs in PB and BM samples that are not consistent with a germline mutational origin (i.e. not close to 50-50, or either VAF is less than 35%). Of these mutations half (3/6) are in the known CH gene DNMT3A. Two were located in CH hotspots (p.R882H, p.R882C), and the other was not previously seen in a study of a large cohort of CH patients (p.A149A). GnomAD showed evidence of germline mutations in the general population at these three DNMT3A loci, but they occur at extremely low frequencies and can be considered very rare. The other three mutations were in ATM, PIGT, and TP53. There were no previous germline mutations found in gnomAD at these loci.

It may be unlikely to have three such rare DNMT3A germline variants in our cohort. It would also be very unlikely that mutations independently occurred in PB and BM cells at these six loci. It is possible that these irregular VAFs may be due to genetic mosaicism. Genetic mosaicism is the presence of two or more genetically different sets of cells in an individual, arising from mutations in progenitor cells. If a somatic mutation occurred in multipotent hematopoietic stem cells (a progenitor to bone marrow and peripheral blood cells) or in an earlier pluripotent progenitor cell during embryonic development, we might observe high VAFs in either PB samples, BM samples, or both for a given individual. However, genetic mosaicism and germline variation are indistinguishable in the available sequencing data.

**References**

1. Kent WJ. BLAT--the BLAST-like alignment tool. *Genome research* 2002; **12**(4): 656-64.

2. Lawrence MS, Stojanov P, Mermel CH, et al. Discovery and saturation analysis of cancer genes across 21 tumour types. *Nature* 2014; **505**(7484): 495-501.

3. Ellrott K, Bailey MH, Saksena G, et al. Scalable Open Science Approach for Mutation Calling of Tumor Exomes Using Multiple Genomic Pipelines. *Cell Syst* 2018; **6**(3): 271-81 e7.

4. Thorvaldsdottir H, Robinson JT, Mesirov JP. Integrative Genomics Viewer (IGV): high-performance genomics data visualization and exploration. *Brief Bioinform* 2013; **14**(2): 178-92.

5. Taylor-Weiner A, Stewart C, Giordano T, et al. DeTiN: overcoming tumor-in-normal contamination. *Nature methods* 2018; **15**(7): 531-4.

6. Carter SL, Cibulskis K, Helman E, et al. Absolute quantification of somatic DNA alterations in human cancer. *Nat Biotechnol* 2012; **30**(5): 413-21.
