# Supplementary material for: Clinical Outcomes and Evolution of Clonal Hematopoiesis in Patients with Newly Diagnosed Multiple Myeloma
Source: Cancer Res Commun. 2023 Dec 18;3(12):2560–71. doi: 10.1158/2767-9764.CRC-23-0093 (PMC10730502; doi:10.1158/2767-9764.CRC-23-0093)
Supplement: Supplementary Figure 1 — Depiction of cell mixtures for the normal (PB) sample and tumor (BM) sample showing effect of tumor purity and tumor-in-normal (TiN) contamination. Relative areas represent DNA fractions of different cell types. [file crc-23-0093-s02.docx]

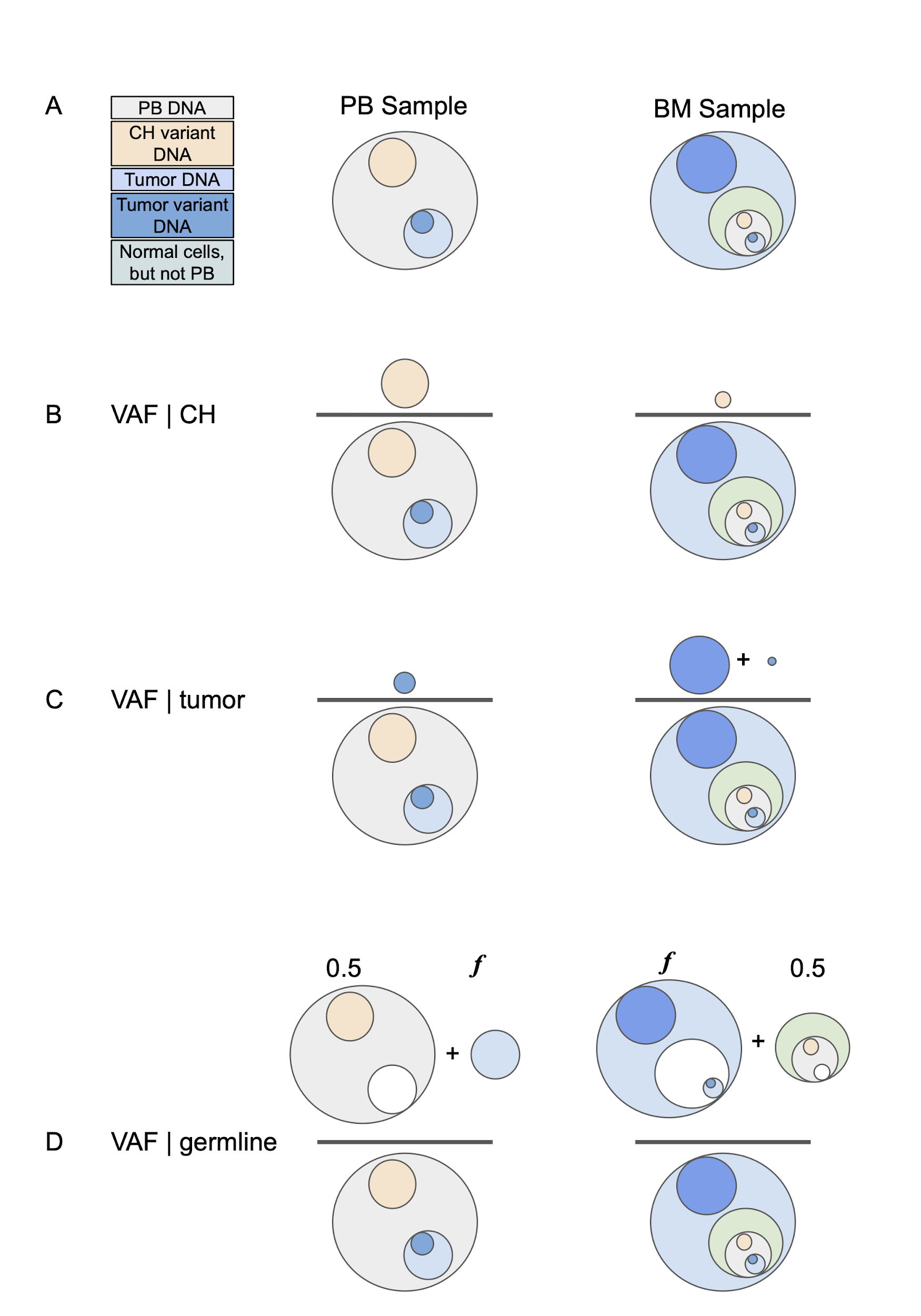


**Supplementary Figure 1.** Depiction of cell mixtures for the normal (PB) sample and tumor (BM) sample showing effect of tumor purity and tumor-in-normal (TiN) contamination. Relative areas represent DNA fractions of different cell types.

A. Overall cell type mixtures. The small image of the BM sample within the PB sample arises from tumor-in-normal contamination, which includes all components of cells within the tumor sample, including non-PB normal cells (green), PB cells (gray), and CH DNA (tan). The BM sample also contains tumor DNA that does not carry the mutation (light blue) and mutated DNA (darker blue). The BM sample contains dominant tumor components (blue) along with normal cells that correspond to tumor purity. The green region bounding the normal cells of the normal sample within the BM sample arises from tumor purity, which includes a component of normal cells that are not PB (green), PB cells (gray), and CH DNA (tan).

B: Components of DNA that are included in the VAF numerator (above the line) for the case of CH mutations for the normal sample (left side) and the tumor sample (right side). The denominators are the all DNA in each respective sample.

C: Components of DNA that are included in the VAF numerator for the case of tumor mutations of the normal sample (left side) and the tumor sample (right side).

D. Components of DNA that are included in the VAF numerator for the case of germline variants (hets) of the normal sample (left side, with VAF=0.5) and the tumor sample (right side, with VAF=*f* not necessarily consistent with 0.5 due to underlying copy number variation).
