# Supplementary material for: Clinical Outcomes and Evolution of Clonal Hematopoiesis in Patients with Newly Diagnosed Multiple Myeloma
Source: Cancer Res Commun. 2023 Dec 18;3(12):2560–71. doi: 10.1158/2767-9764.CRC-23-0093 (PMC10730502; doi:10.1158/2767-9764.CRC-23-0093)
Supplement: Supplementary Figure 2 — Depiction of cell mixtures for PB and BM samples. [file crc-23-0093-s03.docx]

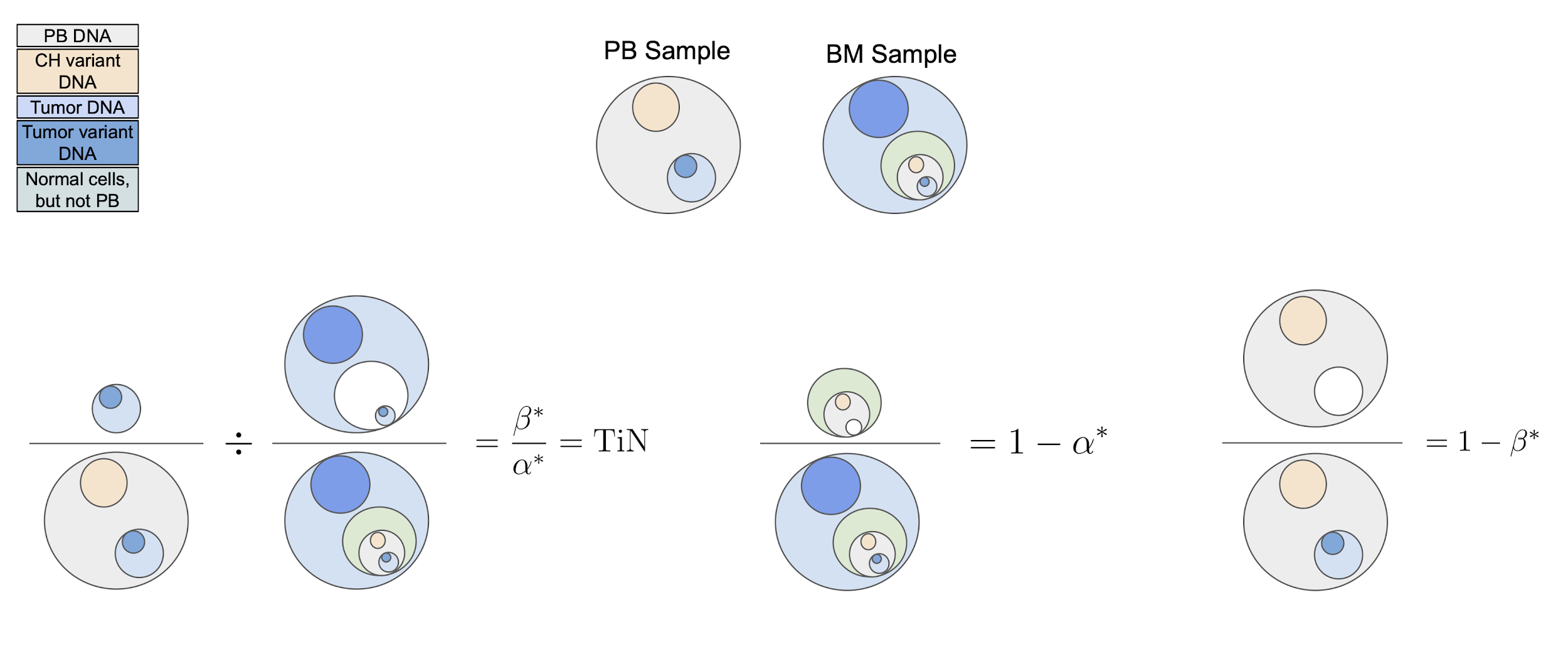


**Supplementary Figure 2.** Depiction of cell mixtures for PB and BM samples and derivations of [
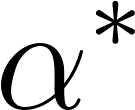
](https://latex-staging.easygenerator.com/eqneditor/editor.php?latex=%5Calpha%5E%7B*%7D#0), [
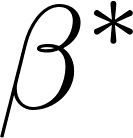
](https://latex-staging.easygenerator.com/eqneditor/editor.php?latex=%5Cbeta%5E%7B*%7D#0), and TiN.
