# Supplementary material for: Clinical Outcomes and Evolution of Clonal Hematopoiesis in Patients with Newly Diagnosed Multiple Myeloma
Source: Cancer Res Commun. 2023 Dec 18;3(12):2560–71. doi: 10.1158/2767-9764.CRC-23-0093 (PMC10730502; doi:10.1158/2767-9764.CRC-23-0093)
Supplement: Supplementary Figure 4 — Effect of IMiDs and CH on overall survival. [file crc-23-0093-s05.docx]

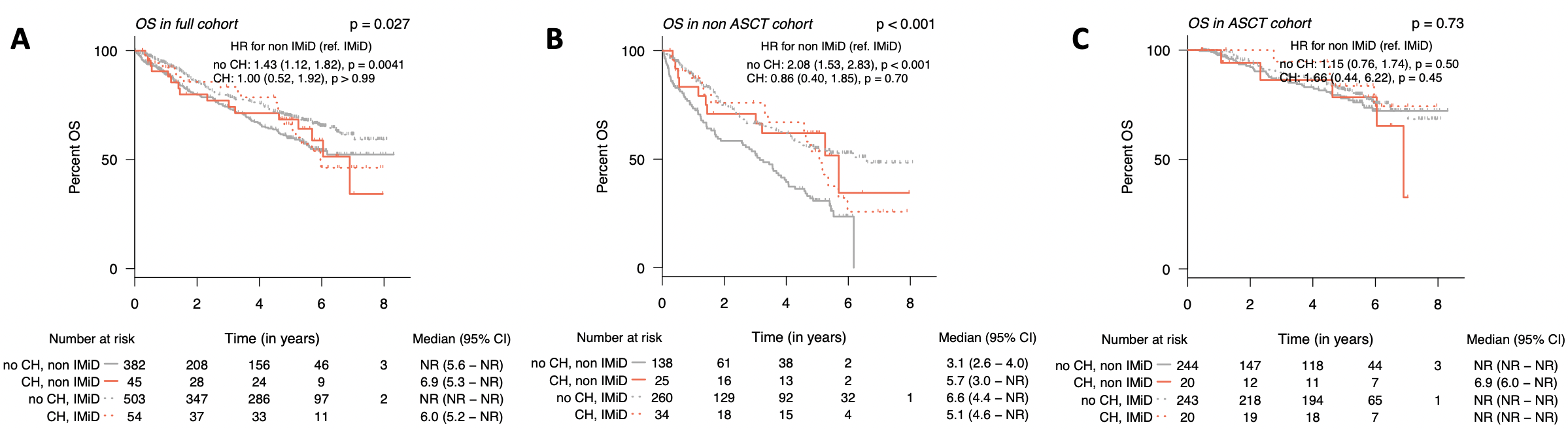


**Supplementary Figure 4. Effect of IMiDs and CH on overall survival.** OS among **(A)** the full cohort, **(B)** non-transplanted and **(C)** transplanted patients with respect to having CH and receiving IMiD-based therapies.
