# Supplementary material for: Clinical Outcomes and Evolution of Clonal Hematopoiesis in Patients with Newly Diagnosed Multiple Myeloma
Source: Cancer Res Commun. 2023 Dec 18;3(12):2560–71. doi: 10.1158/2767-9764.CRC-23-0093 (PMC10730502; doi:10.1158/2767-9764.CRC-23-0093)
Supplement: Supplementary Figure 5 — Temporal changes in CH in MM. [file crc-23-0093-s06.docx]

**Supplementary Figure 5. Temporal changes in CH in MM.** The figure depicts the change in prevalence and VAF of CH clones between two different time points in 13 myeloma patients undergoing treatment. Time point 1 depicts 3 CH mutations, while time point 2 depicts 14 CH mutations. Red lines correspond to patients who underwent ASCT in between the two time points, while black lines indicate that there was no ASCT. Similarly, the straight lines indicate that patients were treated with IMiDs, while the one dotted line signifies a patient that did not receive any IMiD therapy.
