# Supplementary material for: Clinical Outcomes and Evolution of Clonal Hematopoiesis in Patients with Newly Diagnosed Multiple Myeloma
Source: Cancer Res Commun. 2023 Dec 18;3(12):2560–71. doi: 10.1158/2767-9764.CRC-23-0093 (PMC10730502; doi:10.1158/2767-9764.CRC-23-0093)
Supplement: Supplementary Figure 6 — Power to detect CH mutations. [file crc-23-0093-s07.docx]

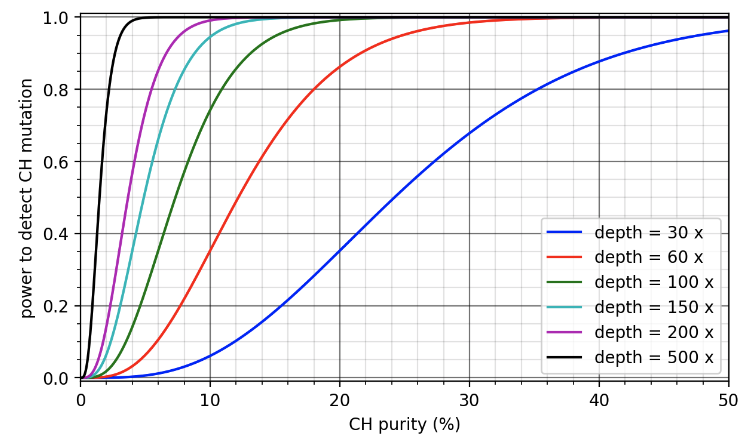


**Supplementary Figure 6. Power to detect CH mutations.** Power to detect any given CH mutation depends on the PB sample purity and the sequencing depth at the site. Here the detection threshold is simply set to 4 or more alternate allele supporting reads. PB sample purity for the percentage of CH cells is shown on the x axis, detection power on the y axis, and line color indicate the sequencing depth at a given site, where blue corresponds to 30x depth, red: 60x, green: 100x, cyan: 150x, purple: 200x, and black: 500x. The MMRF cohort median depth was ~120x between the cyan and green lines.
