# Supplementary material for: Clinical Outcomes and Evolution of Clonal Hematopoiesis in Patients with Newly Diagnosed Multiple Myeloma
Source: Cancer Res Commun. 2023 Dec 18;3(12):2560–71. doi: 10.1158/2767-9764.CRC-23-0093 (PMC10730502; doi:10.1158/2767-9764.CRC-23-0093)
Supplement: Supplementary Table 1 — List of queried genes and variants to determine the presence of CH. [file crc-23-0093-s08.docx]

**Supplementary Table 1. List of queried genes and variants to determine the presence of CH**

| **Gene Name** | **Mutations used for variant calling** |
| --- | --- |
| ASXL1 | Frameshift/nonsense/splice-site in exon 11-12; aa_range: 400-1540 |
| ASXL2 | Frameshift/nonsense/splice-site in exon 11-12 |
| ATM | Frameshift/nonsense/splice-site |
| ATRX | Frameshift/nonsense/splice-site |
| B2M | Frameshift/nonsense/splice-site |
| BCOR | Frameshift/nonsense/splice-site |
| BCORL1 | Frameshift/nonsense/splice-site |
| BIRC3 | Frameshift/nonsense/splice-site in exon 2 |
| BRAF | G464E, G464V, G466E, G466V, G469R, G469E, G469A, G469V, V471F, V472S, L485W, N581S, I582M, I592M, I592V, D594N, D594G, D594V, D594E, F595L, F595S, G596R, L597V, L597S, L597Q, L597R, A598V, V600M, V600L, V600K, V600R, V600E, V600A, V600G, V600D, K601E, K601N, R603*, W604R, W604G, S605G, S605F, S605N, G606E, G606A, G606V, H608R, H608L, G615R, S616P, S616F, L618S, L618W |
| BRCC3 | Frameshift/nonsense/splice-site |
| CALR | Frameshift and aa_range: 352-418 |
| CARD11 | E93D, G123S, G126D, T128M, F130I, R179W, K182N, M183L, K215M, D230N, L232LI, M240MGLNKM, K244T, S250P, S250P, L251P, L251P, V266*, D338G, T353*, D357V, Y361H, M365K, D387V, D387V, D401V, R418S, R423W, E432K, E626K |
| CBL | Missense/frameshift/non-frameshift p.360-430 OR splice site |
| CBLB | Missense p.360-430 |
| CDKN2A | Frameshift/nonsense/splice-site |
| CEBPA | Frameshift/nonsense/splice-site and missense and ExAc<0.001 |
| CHD2 | H620L, F1146L, L1270F |
| CREBBP | Frameshift/nonsense/splice-site, D1435E, R1446L, R1446H, R1446C, Y1450C, P1476R, Y1482H, H1487Y, W1502C, Y1503D, Y1503H, Y1503F, S1680del |
| CSF1R | Frameshift/nonsense, L301F, L301S, Y969C, Y969N, Y969F, Y969H, Y969D |
| CSF3R | Frameshift/nonsense p.618-840 (truncating c.741-791), T615A, T618I |
| CSNK1A1 | E98, D140, missense with ExAC frequency <0.0001 |
| CTC1 | Frameshift/nonsense/splice-site |
| CTCF | Frameshift/nonsense, R377C, R377H, P378A, P378L |
| CUX1 | Frameshift/nonsense/splice-site |
| DDX41 | Frameshift/nonsense/splice-site, missense and aa_range: 360-430 with ExAC_Freq<0.0001 |
| DNMT3A | Frameshift/nonsense/splice-site, F290I, F290C, V296M, P307S, P307R, R326H, R326L, R326C, R326S, G332R, G332E, V339A, V339M, V339G, L344Q, L344P, R366P, R366H, R366G, A368T, A368V, R379H, R379C, I407T, I407N, I407S, F414L, F414S, F414C, A462V, K468R, C497G, C497Y, Q527H, Q527P, Y533C, S535F, C537G, C537R, G543A, G543S, G543C, L547H, L547P, L547F, M548I, M548K, G550R, W581R, W581G, W581C, R604Q, R604W, R635W, R635Q, S638F, G646V, G646E, L653W, L653F, I655N, V657A, V657M, R659H, Y660C, V665G, V665L, M674V, R676W, R676Q, G685R, G685E, G685A, D686Y, D686G, R688H, G699R, G699S, G699D, P700L, P700S, P700R, P700Q, P700T, P700A, D702N, D702Y, V704M, V704G, I705F, I705T, I705S, I705N, G707D, G707V, C710S, C710Y, S714C, V716D, V716F, V716I, N717S, N717I, P718L, R720H, R720G, K721R, K721T, Y724C, R729Q, R729W, R729G, F731C, F731L, F731Y, F731I, F732del, F732C, F732S, F732L, E733G, E733A, F734L, F734C, Y735C, Y735N, Y735S, R736H, R736C, R736P, L737H, L737V, L737F, L737R, A741V, P742P, P743R, P743L, R749C, R749L, R749H, R749G, F751L, F751C, F752del, F752C, F752L, F752I, F752V, W753G, W753C, W753R, L754P, L754R, L754H, F755S, F755I, F755L, M761I, M761V, G762C, V763I, S770L, S770W, S770P, R771Q, F772I, F772V, L773R, L773V, E774K, E774D, E774G, I780T, D781G, R792H, W795C, W795L, G796D, G796V, N797Y, N797H, N797S, P799S, P799R, P799H, R803S, R803W, P804L, P804S, K826R, S828N, K829R, T835M, N838D, K841Q, Q842E, P849L, D857N, W860R, E863D, F868S, G869S, G869V, M880V, S881R, S881I, R882H, R882P, R882C, R882G, A884P, A884V, Q886R, L889P, L889R, G890D, G890R, G890S, V895M, P896L, V897G, V897D, R899L, R899H, R899C, L901R, L901H, P904L, F909C, P904Q, A910P, C911R, C911Y |
| EED | Frameshift/nonsense/splice-site, L240Q, I363M |
| EP300 | Frameshift/nonsense/splice-site, VF1148_1149del, D1399N, D1399Y, P1452L, Y1467N, Y1467H, Y1467C, R1627W, A1629V |
| ETNK1 | N244S, N244T, N244K |
| ETV6 | Frameshift/nonsense/splice-site, missense and aa_range: 338-424 and #ETS domain and ExAC_Freq<0.0001, missense and aa_range: 56-123 and #PNT domain and ExAC_Freq<0.0001 |
| EZH2 | Frameshift/nonsense/splice-site, Q62R, N102S, F145S, F145C, F145Y, F145L, G159R, E164D, R202Q, K238E, E244K, R283Q, H292R, P488S, R497Q, R561H, T568I, K629E, Y641N, Y641H, Y641S, Y641C, Y641F, D659Y, D659G, V674M, A677G, A677V, R679C, R679H, R685C, R685H, A687V, N688I, N688K, H689Y, S690P, I708V, I708T, I708M, E720K, E740K |
| FAM46C | Frameshift/nonsense/splice-site |
| FANCL | Frameshift/nonsense/splice-site |
| FLT3 | frameshift and aa_range: 569-648, nonframeshift and aa_range: 569-700, nonframeshift and aa_range: 800-850, V579A, V592A, V592I, F594L, FY590-591GD, N676, N663, F691, D835Y, D835H, D835E, del835, N841, Y842 |
| GATA1 | Frameshift/nonsense/splice-site, missense and ExAC_Freq<0.0001 |
| GATA2 | Frameshift/nonsense/splice-site, R293Q, N317H, A318T, A318V, A318G, G320D, L321P, L321F, L321V, Q328P, R330Q, R361L, L359V, A372T, R384G, R384K, position:128202118,128202197 |
| GATA3 | Frameshift/nonsense/splice-site ZNF domain, R276W, R276Q, N286T, L348V |
| GNA13 | I34T, G57S, S62F, M68K, Q134R, Y145F, L152F, E167D, Q169H, R264H, E273K, V322G, V362G, L371F |
| GNAS | R201(844)S, R201(844)C, R201(844)H, R201(844)L, Q227(870)K, Q227(870)R, Q227(870)L, Q227(870)H, R374(1017)C |
| GNB1 | K57N, K57M, K57E, K57T, D76, K78, I80T, I80N, K89, N88 |
| HIST1H1E | A158T, A167V, P196S, K202E, K205R |
| IDH1 | Nonframeshift and aa_range: 126-138 , R132C, R132G, R132H, R132L, R132P, R132V, V178I |
| IDH2 | Nonframeshift and aa_range: 134-146, nonframeshift and aa_range: 164-180, R140W, R140Q, R140L, R140G, R172W, R172G, R172K, R172T, R172M, R172N, R172S |
| IKZF1 | Frameshift/nonsense/splice-site |
| IKZF2 | Frameshift/nonsense |
| IKZF3 | Frameshift/nonsense |
| IRF4 | N2S, S18T, I32V, L40V, Q60K, Q60H |
| JAK1 | T478A, T478S, V623A, A634D, L653F, R724H, R724Q, R724P, T782M, L783F, N533D, N533Y, N533S, H538R, K539E, K539L, I540T, I540V, V617F, R683S, R683G, V683, R867, D873, P933, del/ins537-539L, del/ins538-539L, del/ins540-543MK, del/ins540-544MK, del/ins541-543K, del542-543, del543-544, ins11546-547 |
| JAK3 | M511T, M511I, A572V, A572T, A573V, R657Q, V715I, V715A |
| KDM6A | Frameshift/nonsense/splice-site, del419 |
| KIT | frameshift, ins503, V559A, V559D, V559G, V559I, V560D, V560A, V560G, V560E, del560, E561K, del579, P627L, P627T, R634W, K642E, K642Q, V654A, V654E, H697Y, H697D, E761D, K807R, D816H, D816Y, D816F, D816I, D816V, D816H, del551-559 |
| KRAS | G12D, G12A, G12E, G12V, G13D, G13C, G13Y, G13F, G13R, G13A, G13V, G13E, V14I, T58I, G60D, G60A, G60V, Q61K, Q61E, Q61P, Q61R, Q61L, Q61H, K117E, K117N, A146T, A146P, A146V |
| LRRK2 | E155K, I543S |
| LTB | Frameshift/nonsense |
| LUC7L2 | Frameshift/nonsense/splice-site |
| MIR142 | ExAC_Freq<0.0001 |
| MLL | Frameshift/nonsense |
| MLL2 | Frameshift/nonsense |
| MPL | S505G, S505N, S505C, L510P, del513, W515A, W515R, W515K, W515S, W515L, A519T, A519V, Y591D, W515-518KT |
| MRE11A | Frameshift/nonsense/splice-site |
| MYD88 | L265, V217F , S219C, M240T , S251N , P266 , L273P |
| NF1 | Frameshift/nonsense/splice-site |
| NOTCH1 | Missense and aa_range: 617-738 andExAC_Freq<0.0001, missense and aa_range: 500-616 andExAC_Freq<0.0001, frameshift/nonsense and aa_range: 2061-2555 |
| NOTCH2 | Frameshift/nonsense and aa_range: 2010-2471 |
| NPM1 | Frameshift p.W288fs (insertion at c.859_860, 860_861, 862_863, 863_864)) |
| NRAS | G12S, G12R, G12C, G12N, G12P, G12Y, G12D, G12A, G12V, G12E, G13S, G13R, G13C, G13N, G13P, G13Y, G13D, G13A, G13V, G13E, G60E, G60R, Q61R, Q61L, Q61K, Q61P, Q61H, Q61Q, T74P, A146P |
| PDS5B | Frameshift/nonsense/splice-site, R1292Q |
| PDSS2 | Frameshift/nonsense |
| PHF6 | Frameshift/nonsense/splice-site, A40D, M125I, S246Y, F263L, R274Q, C297Y, H302Y, H329L |
| PHIP | Frameshift/nonsense/splice-site |
| PIGA | Frameshift/nonsense, missense and ExAC_Freq<0.0001 |
| PIGT | Frameshift/nonsense |
| PPM1D | Frameshift/nonsense, exon 5 or 6 |
| PRPF40B | Frameshift/nonsense/splice-site, P15H, M58I, P405L, P562S |
| PRPF8 | Frameshift/nonsense/splice-site, M1307I, C1594W, D1598Y, D1598N, D1598V |
| PTEN | Frameshift/nonsense/splice-site, D24G, R47G, F56V, L57W, H61R, K66N, Y68H, C71Y, F81C, Y88C, D92G, D92V, D92E, H93Y, H93D, H93Q, N94I, P95L, I101T, C105F, C105S, D107Y, L112V, H123Y, C124R, C124S, K125E, A126D, K128N, R130G, R130Q, R130L, G132D, I135V, I135K, C136R, C136F, K144Q, A151T, D153Y, D153N, Y155H, Y155C, R159K, R159S, R161K, R161I, G165R, G165E, S170N, S170I, R173C, Y174D, Y177C, H196Y, R234W, G251C, D252Y, F271S, D326G |
| PTEN | Missense and ExAC_Freq<0.0001 |
| PTPN11 | G60V, G60R, G60A, D61Y, D61V, D61G, Y63C, E69K, E69G, E69D, E69Q, F71L, F71K, A72T, A72V, A72D, T73I, E76K, E76Q, E76M, E76A, E76G, E139G, E139D, N308D, N308T, N339S, P491L, S502P, S502A, S502L, G503V, G503G, G503A, G503E, Q506P, T507A, T507K, missense and ExAC_Freq<0.0001 |
| RAD21 | Frameshift/nonsense/splice-site, R65Q, H208R, Q474R |
| RIT1 | S35, A57, F82, G95, A77, E81, T83, Y89, M90 |
| RPL11 | Frameshift/nonsense, missense and ExAC_Freq<0.0001 |
| RPS7 | Frameshift/nonsense, missense and ExAC_Freq<0.0001 |
| RUNX1 | Frameshift/nonsense/splice-site, S73F, H78Q, H78L, R80C, R80P, R80H, L85Q, P86L, P86H, S114L, D133Y, L134P, R135G, R135K, R135S, R139Q, R142S, A165V, R174Q, R177L, R177Q, A224T, D171G, D171V, D171N, R205W, R223C, missense and ExAC_Freq<0.0001 |
| SETBP1 | D868N, D868T, S869N, G870S, I871T, D880N, D880Q |
| SETD2 | Frameshift/nonsense/splice-site, V1190M |
| SETDB1 | Frameshift/nonsense, K715E |
| SF1 | Frameshift/nonsense/splice-site, T454M, Y476C, A508G |
| SF3A1 | Frameshift/nonsense/splice-site, A57S, M117I, K166T, Y271C, G347V, R387W, R387Q, E592K, E622D, Y623C, R625L, R625C, R625G, H662Q, H662D, T663I, K666N, K666T, K666E, K666R, K700E, V701F, A708T, G740R, G740E, A744P, D781G, E783K, R831Q, L833F, E862K, R957Q |
| SFRS2 | Y44H, P95H, P95L, P95T, P95R, P95A, P107H, P95fs |
| SH2B3 | Frameshift/nonsense, missense and aa_range: 195-307, missense and aa_range: 364-441 |
| SMC1A | Frameshift/nonsense/splice-site, K190T, R586W, M689V, R807H, R1090H, R1090C |
| SMC3 | Frameshift/nonsense/splice-site, R155I, Q367E, D392V, K571R, R661P, G662C |
| SRSF2 | Frameshift and aa_range: 85-100, nonframeshift and aa_range: 85-100, P95 |
| STAG1 | Frameshift/nonsense/splice-site, H1085Y |
| STAG2 | Frameshift/nonsense/splice-site |
| STAT3 | Missense/nonframeshift and aa_range:584-674 |
| STAT5B | missense/nonframeshift and aa_range:593-670 |
| SUZ12 | Frameshift/nonsense |
| TERC | ExAC_Freq<0.0001 |
| TERT | Frameshift/nonsense, missense and ExAC_Freq<0.0001, nonframeshift and ExAC_Freq<0.0001 |
| TET2 | Frameshift/nonsense/splice-site, missense/nonframeshift mutations in catalytic domains (p.1104-1481 and 1843-2002) |
| TNFAIP3 | Frameshift/nonsense, D117V, M476I, P574I |
| TP53 | Frameshift/nonsense/splice-site, S46F, G105C, G105R, G105D, G108S, G108C, R110L, R110C, T118A, T118R, T118I, S127F, S127Y, L130V, L130F, K132Q, K132E, K132W, K132R, K132M, K132N, F134V. F134L, F134S, C135W, C135S, C135F, C135G, C135Y, Q136K, Q136E, Q136P, Q136R, Q136L, Q136H, A138P, A138V, A138A, A138T, T140I, C141R, C141G, C141A, C141Y, C141S, C141F, C141W, V143M, V143A, V143E, L145Q, W146C, W146L, L145R, V147G, P151T, P151A, P151S, P151H, P151R, P152S, P152R, P152L, T155P, T155A, V157F, R158H, R158L, A159V, A159P, A159S, A159D, A161T, A161D, Y163N, Y163H, Y163D, Y163S, Y163C, K164E, K164M, K164N, K164P, H168Y, H168P, H168R, H168L, H168Q, M169I, M169T, M169V, E171K, E171Q, E171G, E171A, E171V, E171D, V172D, V173M, V173L, V173G, R174W, R175G, R175C, R175H, C176R, C176G, C176Y, C176F, C176S, P177R, P177R, P177L, H178D, H178P, H178Q, H179Y, H179R, H179Q, R181C, R181Y, D186G, G187S, P190L, P190T, H193N, H193P, H193L, H193R, L194F, L194R, I195F, I195N, I195Tl R196Pl V197L, G199V, Y205N, Y205C, Y205H, D208V, R213Q, R213P, R213L, R213Q, H214D, H214R, S215G, S215I, S215R, V216M, V217G, Y220N, Y220H, Y220S, Y220C, E224D, I232F, I232N, I232T, I232S, Y234N, Y234H, Y234S, Y234C, Y236N, Y236H, Y236C, M237V, M237K, M237I, C238R, C238G, C238Y, C238W, N239T, N239S, S241Y, S241C, S241F, C242G, C242Y, C242S, C242F, G244S, G244C, G244D, G245S, G245R, G245C, G245D, G245A, G245V, G245S, M246V, M246K, M246R, M246I, N247I, R248W, R248G, R248Q, R249G, R249W, R249T, R249M, P250L, I251N, L252P, I254S, I255F, I255N, I255S, L257Q, L257P, E258K, E258Q, D259Y, S261T, G262D, G262V, L265P, G266R, G266E, G266V, R267W, R267Q, R267P, E271K, V272M, V272L, R273S, R273G, R273C, R273H, R273P, R273L, V274F, V274D, V274A, V274G, V274L, C275Y, C275S, C275F, A276P, C277F, C277Y, P278T, P278A, P278S, P278H, P278R, P278L, G279E, R280G, R280K, R280T, R280I, R280S, D281N, D281H, D281Y, D281G, D281E, R282G, R282W, R282Q, R282P, E285K, E285V, E286G, E286V, E286K, K320N, L330R, G334V, R337C, R337L, A347T, L348F, T377P, missense and ExAC_Freq<0.0001 |
| TRAF3 | Frameshift/nonsense |
| U2AF1 | Nonframeshift and aa_range: 30-38, nonframeshift and aa_range: 153-161, D14G, S34F, S34Y, R35L, R156H, R156Q, Q157R, Q157P |
| U2AF2 | R18W, Q143L, M144I, L187V, Q190L, nonframeshift and ExAC_Freq<0.0001 |
| VPS45 | Frameshift/nonsense, missense/nonframeshift and ExAC_Freq<0.0001 |
| WT1 | Frameshift/nonsense/splice-site |
| XPO1 | E571A, E571K |
| ZRSR2 | Frameshift/nonsense/splice-site, R126P, E133G, C181F, H191Y, I202N, F239V, F239Y, N261Y, C280R, C302R, C326R, H330R, N382K |
