# Supplementary material for: Clinical Outcomes and Evolution of Clonal Hematopoiesis in Patients with Newly Diagnosed Multiple Myeloma
Source: Cancer Res Commun. 2023 Dec 18;3(12):2560–71. doi: 10.1158/2767-9764.CRC-23-0093 (PMC10730502; doi:10.1158/2767-9764.CRC-23-0093)
Supplement: Supplementary Table 2 — List of genes in the targeted sequencing panel. [file crc-23-0093-s09.docx]

**Supplementary Table 2. List of genes in the targeted sequencing panel**

| Genes with coding exons covered (n=540) | | | | | | | |
| --- | --- | --- | --- | --- | --- | --- | --- |
| ABL1 | C11ORF30/EMSY | DOT1L | GNAQ | LCK | NSD1 | RAD51 | STAT4 |
| ABL2 | C3ORF70 | EIF2S2 | GNAS | LCTL | NTN4 | RAF1 | STAT5B |
| ACO1 | CACNA1D | ELF3 | GNB1 | LIFR | NTRK1 | RALGDS | STK11 |
| ACVR1B | CALR | EML4 | GNPTAB | LMO1 | NTRK2 | RARA | STK19 |
| ACVR2B | CAP2 | EP300 | GOT1 | LPP | NTRK3 | RASA1 | STX2 |
| ADNP | CARD11 | EPHA2 | GPC3 | LRP1B | NUMA1 | RBM10 | SUFU |
| AJUBA | CASP8 | EPHA3 | GPR124 | LTF | NUP210L | RECQL4 | SYK |
| AKAP9 | CBFB | EPHA5 | GPS2 | MAF | NUP214 | REL | TAL1 |
| AKT1 | CBL | EPHB1 | GRIN2A | MAFB | NUP93 | RET | TAP1 |
| AKT2 | CCDC120 | EPHB4 | GSK3B | MALT1 | NUP98 | RHEB | TBC1D12 |
| AKT3 | CCDC6 | ERBB2 | GUSB | MAML2 | ODAM | RHOA | TBL1XR1 |
| ALK | CCND1 | ERBB3 | H3F3A | MAP2K1 | OMA1 | RICTOR | TBX3 |
| ALKBH6 | CCND2 | ERBB4 | H3F3B | MAP2K2 | OR4A16 | RIT1 | TCEB1 |
| ALPK2 | CCND3 | ERCC2 | HGF | MAP2K4 | OR52N1 | RNASEL | TCF12 |
| AMER1/FAM123B | CCNE1 | ERCC3 | HIST1H1E | MAP3K1 | OTUD7A | RNF43 | TCF3 |
| ANK3 | CD1D | ERCC4 | HIST1H3B | MAP4K3 | PAK3 | ROS1 | TCF7L2 |
| APC | CD70 | ERCC5 | HIST1H4E | MBD1 | PALB2 | RPL5 | TCL1A |
| APOL2 | CD79A | ERG | HLA-A | MCL1 | PAPD5 | RPS15 | TCP11L2 |
| AR | CD79B | ESR1 | HLA-B | MDM2 | PAX3 | RPS2 | TDRD10 |
| ARAF | CDC27 | ETS1 | HLF | MDM4 | PAX5 | RPS6KA2 | TET2 |
| ARFRP1 | CDC73 | ETV1 | HMGA2 | MED12 | PAX7 | RPTOR | TFE3 |
| ARHGHP35 | CDH1 | ETV4 | HNF1A | MED23 | PAX8 | RSBN1L | TGFBR2 |
| ARID1A | CDH11 | EXT1 | HOOK3 | MEF2B | PBRM1 | RUNX1 | THBS1 |
| ARID1B | CDK12 | EXT2 | HRAS | MEN1 | PBX1 | RXRA | TIMM17A |
| ARID2 | CDK4 | EZH1 | HSP90AB1 | MET | PCBP1 | SACS | TIMP3 |
| ARID5B | CDK6 | EZH2 | IDH1 | MGA | PDAP1 | SBDS | TLX1 |
| ARNT | CDK8 | EZR | IDH2 | MICALCL | PDCD2L | SDHA | TNF |
| ASXL1 | CDKN1A | FAM166A | IGF1R | MITF | PDE4DIP | SDHAF2/SDH5 | TNFAIP3 |
| ASXL2 | CDKN1B | FAM46C | IGF2R | MLH1 | PDGFB | SDHB | TNFRSF14 |
| ATF1 | CDKN2B | FANCA | IKBKE | MLLT10 | PDGFRA | SDHC | TOP1 |
| ATM | CEBPA | FANCC | IL2 | MN1 | PDGFRB | SDHD | TP53BP1 |
| ATP1A1 | CEP76 | FANCD2 | IL21R | MORC4 | PDK1 | SELP | TPR |
| ATP2B3 | CHD4 | FANCE | IL6ST | MPL | PDSS2 | SEPT12 | TPX2 |
| ATP5B | CHD8 | FANCF | IL7R | MPO | PER1 | SEPT4 | TRAF7 |
| ATR | CHEK1 | FANCG | ING1 | MRE11A | PHF6 | SERPINB13 | TRIM23 |
| ATRX | CHEK2 | FANCL | ING4 | MSH2 | PHLPP2 | SETBP1 | TRIM24 |
| AURKA | CIC | FAT1 | INHBA | MSH6 | PHOX2B | SETD2 | TRIM33 |
| AURKB | CNBD1 | FGF10 | INPPL1 | MTOR | PIK3CA | SETDB1 | TRIP11 |
| AXIN1 | CNKSR1 | FGF14 | INTS12 | MUC1 | PIK3CG | SEF3B1 | TRRAP |
| AXIN2 | COL1A1 | FGF19 | IPO7 | MUC17 | PIK3R1 | SFRS2 | TSHR |
| AXL | COL5A1 | FGF23 | IRF4 | MUTYH | PIK3R2 | SGK1 | TTLL9 |
| AZGP1 | COL5A3 | FGF3 | IRF6 | MXRA5 | PIM1 | SIRT4 | TXNDC8 |
| B2M | CREB1 | FGF4 | IRS2 | MYB | PLAG1 | SKP2 | U2AF1 |
| BAI3 | CREBBP | FGF6 | ITGB3 | MYC | PLCG2 | SLC1A3 | UBR5 |
| BAP1 | CRKL | FGFBP1 | ITGB7 | MYCL1 | PML | SLC26A3 | WAS |
| BARD1 | CRLF2 | FGFR4 | ITPKB | MYCN | PMS1 | SLC44A3 | WASF3 |
| BCL10 | CSF1R | FH | JAK1 | MYD88 | PMS2 | SLC4A5 | WHSC1 |
| BCL11A | CTCF | FHIT | JAK2 | MYH11 | POLE | SMAD2 | WISP3 |
| BCL11B | CTNNA1 | FLCN | JAK3 | MYH9 | POT1 | SMAD3 | WRN |
| BCL2 | CTNNB1 | FLG | JUN | MYOCD | POU2AF1 | SMARCA4 | XIRP2 |
| BCL2L2 | CUL4B | FLI1 | KAT6A | NBPF1 | POU2F2 | SMARCB1 | XPA |
| BCL3 | CUX1 | FLT1 | KCNJ5 | NBS1 | POU5F1 | SMC1A | XPC |
| BCL6 | CYLD | FLT3 | KDM5A | NCOA1 | PPARG | SMC3 | XPO1 |
| BCL9 | DAXX | FLT4 | KDM5C | NCOA2 | PPM1D | SMO | ZFHX3 |
| BCLAF1 | DCC | FOXA1 | KDM6A | NCOA3 | PPP2R1A | SNX25 | ZNF180 |
| BCOR | DDB2 | FOXL2 | KDR | NCOA4 | PPP6C | SOCS1 | ZNF217 |
| BCORL1 | DDIT3 | FOXO1 | KEAP1 | NCOR1 | PRDM1 | SOS1 | ZNF384 |
| BCR | DDR2 | FOXO3 | KEL | NF2 | PREX2 | SOX10 | ZNF471 |
| BHMT2 | DDX3X | FOXP1 | KIT | NFE2L2 | PRKAR1A | SOX11 | ZNF483 |
| BIRC3 | DDX5 | FOXQ1 | KLF4 | NFKB1 | PRKDC | SOX17 | ZNF521 |
| BLM | DEK | FRMD7 | KLF6 | NFKB2 | PTCH1 | SOX2 | ZNF620 |
| BMPR1A | DIAPH1 | FUBP1 | KLHL6 | NFKBIA | PTCH2 | SOX9 | ZNF703 |
| BRAF | DICER1 | GATA1 | KLHL8 | NIN | PTPN11 | SPEN | ZNF750 |
| BRD3 | DIS3 | GATA2 | KMT2A/MLL | NKX2-1 | QKI | SPOP | ZRANB3 |
| BRE | DNAH12 | GATA3 | KMT2B/MLL2 | NOTCH1 | RAB40A | SRC |  |
| BRIP1 | DNER | GID4 | KMT2C/MLL3 | NOTCH2 | RAC1 | SRSF2 |  |
| BTK | DNMT1 | GNA11 | KMT2D/MLL4 | NPM1 | RAD21 | SSX1 |  |
| BUB1B | DNT3A | GNA13 | KRAS | NRAS | RAD50 | STAT3 |  |
| Genes with coding and non-coding regions covered for identifying structural events (n=22) | | | | | | | |
| BRCA1 | CDKN2C | FGFR1 | IKZF1 | PTPRD | STAG2 | TSC1 | WT1 |
| BRCA2 | EGFR | FGFR2 | NF1 | RB1 | TP53 | TSC2 |  |
| CDKN2A | FBXW7 | FGFR3 | PTEN | SMAD4 | TRAF3 | VHL |  |
| Genes with specific structurally involved regions covered for identifying specific structural events (n=22) | | | | | | | |
| ALK | ERG | HMGA2 | NTRK1 | PLAG1 | ROS1 | SS18 | TMPRSS2 |
| BCR | EWSR1 | JAK2 | PDGFB | RARA | RSPO2 | TCF3 |  |
| BRAF | FUS | MAML2 | PDGFRA | RET | RSPO3 | TFE3 |  |
