# Supplementary material for: Clinical Outcomes and Evolution of Clonal Hematopoiesis in Patients with Newly Diagnosed Multiple Myeloma
Source: Cancer Res Commun. 2023 Dec 18;3(12):2560–71. doi: 10.1158/2767-9764.CRC-23-0093 (PMC10730502; doi:10.1158/2767-9764.CRC-23-0093)
Supplement: Supplementary Table 3 — List of 145 candidate mutations in the baseline samples classified as either CH, Tumor or Germline. [file crc-23-0093-s10.docx]

**Supplementary Table 3. List of 145 candidate mutations in the baseline samples classified as either CH, Tumor or Germline**

| Gene | Chromosome | Start Position | End Position | Variant Classification | Variant Type | Reference Allele | Tumor Seq Allele2 | Codon Change | Protein Change | Allele Frequency | Sample | Mutation Origin |
| --- | --- | --- | --- | --- | --- | --- | --- | --- | --- | --- | --- | --- |
| ASXL1 | 20 | 31023208 | 31023208 | Nonsense | SNP | G | A | c.(2692-2694)tGg>tAg | p.W898* | 0.046154 | MMRF_1037_1_PB | CH |
| ASXL1 | 20 | 31022403 | 31022425 | Frameshift | DEL | CACCACTGCCATAGAGAGGCGGC | - | c.(1888-1911)caccactgccatagagaggcggccfs | p.HHCHREAA630fs | 0.05555556 | MMRF_1045_1_PB | CH |
| ASXL1 | 20 | 31021610 | 31021610 | Nonsense | SNP | G | T | c.(1609-1611)Gaa>Taa | p.E537* | 0.053191 | MMRF_1423_1_PB | CH |
| ASXL1 | 20 | 31022288 | 31022288 | Nonsense | SNP | C | A | c.(1771-1773)taC>taA | p.Y591* | 0.086957 | MMRF_1499_2_PB | CH |
| ASXL1 | 20 | 31021211 | 31021211 | Nonsense | SNP | C | T | c.(1210-1212)Cga>Tga | p.R404* | 0.108696 | MMRF_2014_1_PB | CH |
| ASXL1 | 20 | 31023159 | 31023159 | Nonsense | SNP | C | T | c.(2644-2646)Caa>Taa | p.Q882* | 0.029126 | MMRF_2174_1_PB | CH |
| ASXL1 | 20 | 31022853 | 31022853 | Nonsense | SNP | C | T | c.(2338-2340)Cag>Tag | p.Q780* | 0.071429 | MMRF_2429_1_PB | CH |
| ASXL1 | 20 | 31023540 | 31023541 | Frameshift | DEL | CT | - | c.(3025-3027)ctcfs | p.L1009fs | 0.21428571 | MMRF_2526_1_PB | CH |
| ATM | 11 | 108224493 | 108224493 | Splice site | SNP | G | A | c.(8671-8673)gGt>gAt | p.G2891D | 0.11236 | MMRF_1318_2_PB | CH |
| ATM | 11 | 108141874 | 108141874 | Splice site | SNP | G | A | c.e20+1 |  | 0.037037 | MMRF_1560_1_PB | Tumor |
| ATM | 11 | 108115562 | 108115562 | Frameshift | DEL | C | - | c.(709-711)actfs | p.T237fs | 0.29166667 | MMRF_1588_1_PB | CH |
| ATM | 11 | 108141792 | 108141792 | Splice site | SNP | A | G | c.(2839-2841)tAt>tGt | p.Y947C | 0.30303 | MMRF_2160_1_PB | Germline |
| ATM | 11 | 108137946 | 108137946 | Nonsense | SNP | G | T | c.(2515-2517)Gaa>Taa | p.E839* | 0.059603 | MMRF_2244_1_PB | CH |
| ATM | 11 | 108124587 | 108124587 | Nonsense | SNP | G | T | c.(1945-1947)Gaa>Taa | p.E649* | 0.226667 | MMRF_2650_1_PB | Tumor |
| ATM | 11 | 108115595 | 108115595 | Missense | SNP | G | A | c.(742-744)cGa>cAa | p.R248Q | 0.088608 | MMRF_2650_1_PB | Tumor |
| BCORL1 | X | 129159151 | 129159151 | Missense | SNP | G | A | c.(3874-3876)cGa>cAa | p.R1292Q | 0.066667 | MMRF_1992_1_PB | Tumor |
| BRAF | 7 | 140453155 | 140453155 | Missense | SNP | C | T | c.(1780-1782)Gat>Aat | p.D594N | 0.098039 | MMRF_1778_1_PB | Tumor |
| BRAF | 7 | 140453136 | 140453136 | Missense | SNP | A | T | c.(1798-1800)gTg>gAg | p.V600E | 0.195652 | MMRF_1897_1_PB | Tumor |
| DNMT3A | 2 | 25457243 | 25457243 | Missense | SNP | G | A | c.(2644-2646)Cgc>Tgc | p.R882C | 0.02439 | MMRF_1038_1_PB | CH |
| DNMT3A | 2 | 25468888 | 25468888 | Splice site | SNP | C | A | c.e12+1 |  | 0.164384 | MMRF_1079_2_PB | CH |
| DNMT3A | 2 | 25458595 | 25458595 | Missense | SNP | A | G | c.(2587-2589)Tgg>Cgg | p.W863R | 0.11 | MMRF_1143_2_PB | CH |
| DNMT3A | 2 | 25470990 | 25470990 | Frameshift | DEL | A | - | c.(769-771)actfs | p.T257fs | 0.19047619 | MMRF_1179_1_PB | CH |
| DNMT3A | 2 | 25464464 | 25464464 | Nonsense | SNP | G | C | c.(2047-2049)taC>taG | p.Y683* | 0.030303 | MMRF_1307_1_PB | CH |
| DNMT3A | 2 | 25470484 | 25470484 | Nonsense | SNP | C | T | c.(988-990)tgG>tgA | p.W330* | 0.07907 | MMRF_1355_2_PB | CH |
| DNMT3A | 2 | 25458575 | 25458575 | Splicesite | SNP | C | T | c.e22+1 |  | 0.060606 | MMRF_1356_1_PB | CH |
| DNMT3A | 2 | 25467466 | 25467466 | Missense | SNP | C | A | c.(1609-1611)tGc>tTc | p.C537F | 0.10596 | MMRF_1388_1_PB | CH |
| DNMT3A | 2 | 25467208 | 25467208 | Splice site | SNP | C | A | c.e15-1 |  | 0.160305 | MMRF_1447_1_PB | CH |
| DNMT3A | 2 | 25457176 | 25457176 | Missense | SNP | G | T | c.(2710-2712)cCg>cAg | p.P904Q | 0.166667 | MMRF_1451_1_PB | CH |
| DNMT3A | 2 | 25457242 | 25457242 | Missense | SNP | C | T | c.(2644-2646)cGc>cAc | p.R882H | 0.492958 | MMRF_1539_1_PB | Germline |
| DNMT3A | 2 | 25470029 | 25470029 | Splice site | SNP | T | A | c.e9-2 |  | 0.037975 | MMRF_1556_1_PB | CH |
| DNMT3A | 2 | 25459806 | 25459806 | Splice site | SNP | T | C | c.(2476-2478)aAg>aGg | p.K826R | 0.04918 | MMRF_1572_1_PB | CH |
| DNMT3A | 2 | 25468935 | 25468935 | Splice site | SNP | T | C | c.e12-2 |  | 0.05 | MMRF_1579_1_PB | CH |
| DNMT3A | 2 | 25458696 | 25458696 | Splice site | SNP | T | C | c.e22-2 |  | 0.064516 | MMRF_1580_1_PB | CH |
| DNMT3A | 2 | 25467134 | 25467134 | Frameshift | DEL | A | - | c.(1741-1743)tggfs | p.W581fs | 0.38947368 | MMRF_1644_1_PB | CH |
| DNMT3A | 2 | 25457243 | 25457243 | Missense | SNP | G | A | c.(2644-2646)Cgc>Tgc | p.R882C | 0.203125 | MMRF_1645_1_PB | Germline |
| DNMT3A | 2 | 25470535 | 25470535 | Nonsense | SNP | C | T | c.(937-939)tgG>tgA | p.W313* | 0.09816 | MMRF_1671_1_PB | CH |
| DNMT3A | 2 | 25467132 | 25467132 | Missense | SNP | C | A | c.(1741-1743)tgG>tgT | p.W581C | 0.089109 | MMRF_1750_1_PB | CH |
| DNMT3A | 2 | 25467509 | 25467509 | Nonsense | SNP | C | A | c.(1567-1569)Gag>Tag | p.E523* | 0.038835 | MMRF_1831_1_PB | CH |
| DNMT3A | 2 | 25470029 | 25470029 | Splice site | SNP | T | A | c.e9-2 |  | 0.024691 | MMRF_1927_1_PB | CH |
| DNMT3A | 2 | 25469028 | 25469028 | Splice site | SNP | C | T | c.e11+1 |  | 0.068182 | MMRF_1997_1_PB | CH |
| DNMT3A | 2 | 25464578 | 25464578 | Splice site | SNP | T | C | c.e17-2 |  | 0.206897 | MMRF_2007_1_PB | CH |
| DNMT3A | 2 | 25469095 | 25469095 | Nonsense | SNP | T | A | c.(1363-1365)Aaa>Taa | p.K455* | 0.018349 | MMRF_2085_2_PB | CH |
| DNMT3A | 2 | 25505311 | 25505311 | Splice site | SNP | C | T | c.(445-447)gcG>gcA | p.A149A | 0.583333 | MMRF_2153_1_PB | Germline |
| DNMT3A | 2 | 25457242 | 25457242 | Missense | SNP | C | T | c.(2644-2646)cGc>cAc | p.R882H | 0.102941 | MMRF_2153_1_PB | CH |
| DNMT3A | 2 | 25458619 | 25458619 | Missense | SNP | T | C | c.(2554-2556)Atg>Gtg | p.M852V | 0.060976 | MMRF_2166_1_PB | CH |
| DNMT3A | 2 | 25467134 | 25467134 | Missense | SNP | A | T | c.(1741-1743)Tgg>Agg | p.W581R | 0.057971 | MMRF_2176_1_PB | CH |
| DNMT3A | 2 | 25470556 | 25470556 | Nonsense | SNP | C | T | c.(916-918)tgG>tgA | p.W306* | 0.040323 | MMRF_2181_1_PB | CH |
| DNMT3A | 2 | 25457242 | 25457242 | Missense | SNP | C | T | c.(2644-2646)cGc>cAc | p.R882H | 0.157895 | MMRF_2226_1_PB | CH |
| DNMT3A | 2 | 25457176 | 25457176 | Missense | SNP | G | A | c.(2710-2712)cCg>cTg | p.P904L | 0.176471 | MMRF_2239_1_PB | CH |
| DNMT3A | 2 | 25463182 | 25463182 | Nonsense | SNP | G | A | c.(2311-2313)Cga>Tga | p.R771* | 0.104167 | MMRF_2257_1_PB | CH |
| DNMT3A | 2 | 25467475 | 25467475 | Missense | SNP | T | G | c.(1600-1602)cAg>cCg | p.Q534P | 0.024631 | MMRF_2290_1_PB | CH |
| DNMT3A | 2 | 25463182 | 25463182 | Nonsense | SNP | G | A | c.(2311-2313)Cga>Tga | p.R771* | 0.042553 | MMRF_2352_1_PB | CH |
| DNMT3A | 2 | 25470920 | 25470920 | Nonsense | SNP | C | A | c.(841-843)Gag>Tag | p.E281* | 0.111111 | MMRF_2461_1_PB | CH |
| DNMT3A | 2 | 25458627 | 25458627 | Missense | SNP | G | A | c.(2545-2547)cCt>cTt | p.P849L | 0.127451 | MMRF_2477_1_PB | CH |
| DNMT3A | 2 | 25457176 | 25457176 | Missense | SNP | G | T | c.(2710-2712)cCg>cAg | p.P904Q | 0.166667 | MMRF_2515_1_PB | CH |
| DNMT3A | 2 | 25467497 | 25467497 | Nonsense | SNP | G | A | c.(1579-1581)Cag>Tag | p.Q527* | 0.040498 | MMRF_2572_1_PB | CH |
| DNMT3A | 2 | 25470620 | 25470620 | Splice site | SNP | T | G | c.e8-2 |  | 0.12987 | MMRF_2665_1_PB | CH |
| DNMT3A | 2 | 25467449 | 25467449 | Missense | SNP | C | A | c.(1627-1629)Ggc>Tgc | p.G543C | 0.180212 | MMRF_2667_1_PB | CH |
| DNMT3A | 2 | 25457243 | 25457243 | Missense | SNP | G | A | c.(2644-2646)Cgc>Tgc | p.R882C | 0.196262 | MMRF_2691_1_PB | CH |
| DNMT3A | 2 | 25468188 | 25468188 | Frameshift | DEL | G | - | c.(1486-1488)tccfs | p.S496fs | 0.3028169 | MMRF_2699_1_PB | CH |
| DNMT3A | 2 | 25470464 | 25470464 | Missense | SNP | G | A | c.(1009-1011)tCa>tTa | p.S337L | 0.059701 | MMRF_2715_1_PB | CH |
| DNMT3A | 2 | 25457242 | 25457242 | Missense | SNP | C | T | c.(2644-2646)cGc>cAc | p.R882H | 0.065574 | MMRF_2728_1_PB | CH |
| DNMT3A | 2 | 25457243 | 25457243 | Missense | SNP | G | A | c.(2644-2646)Cgc>Tgc | p.R882C | 0.070423 | MMRF_2742_1_PB | CH |
| DNMT3A | 2 | 25468935 | 25468935 | Splice site | SNP | T | C | c.e12-2 |  | 0.043103 | MMRF_2796_1_PB | CH |
| DNMT3A | 2 | 25467448 | 25467448 | Missense | SNP | C | A | c.(1627-1629)gGc>gTc | p.G543V | 0.029126 | MMRF_2810_1_PB | CH |
| DNMT3A | 2 | 25458696 | 25458696 | Splice site | SNP | T | C | c.e22-2 |  | 0.051948 | MMRF_2838_1_PB | CH |
| DNMT3A | 2 | 25467493 | 25467493 | Missense | SNP | T | C | c.(1582-1584)tAc>tGc | p.Y528C | 0.045181 | MMRF_2838_1_PB | CH |
| DNMT3A | 2 | 25458575 | 25458575 | Splice site | SNP | C | T | c.e22+1 |  | 0.103093 | MMRF_2853_1_PB | CH |
| ETV6 | 12 | 11905424 | 11905424 | Missense | SNP | C | T | c.(73-75)cCg>cTg | p.P25L | 0.347826 | MMRF_1364_1_PB | CH |
| ETV6 | 12 | 12037414 | 12037414 | Missense | SNP | C | T | c.(1045-1047)Ctt>Ttt | p.L349F | 0.150327 | MMRF_2106_1_PB | Tumor |
| EZH2 | 7 | 148507424 | 148507424 | Splice site | SNP | C | A | c.e17+1 |  | 0.025381 | MMRF_1722_1_PB | CH |
| GNB1 | 1 | 1747229 | 1747229 | Missense | SNP | T | C | c.(169-171)Aag>Gag | p.K57E | 0.081633 | MMRF_1286_1_PB | CH |
| GNB1 | 1 | 1747229 | 1747229 | Missense | SNP | T | C | c.(169-171)Aag>Gag | p.K57E | 0.103896 | MMRF_1447_1_PB | CH |
| JAK2 | 9 | 5073770 | 5073770 | Missense | SNP | G | T | c.(1849-1851)Gtc>Ttc | p.V617F | 0.25974 | MMRF_1652_2_PB | CH |
| JAK3 | 19 | 17937558 | 17937558 | Missense | SNP | A | C | c.(3367-3369)ttT>ttG | p.F1123L | 0.063725 | MMRF_1781_1_PB | CH |
| KRAS | 12 | 25398281 | 25398281 | Missense | SNP | C | T | c.(37-39)gGc>gAc | p.G13D | 0.029661 | MMRF_1431_1_PB | CH |
| KRAS | 12 | 25398281 | 25398281 | Missense | SNP | C | G | c.(37-39)gGc>gCc | p.G13A | 0.072829 | MMRF_2106_1_PB | Tumor |
| NF1 | 17 | 29677227 | 29677227 | Nonsense | SNP | C | T | c.(7348-7350)Cga>Tga | p.R2450* | 0.261261 | MMRF_2691_1_PB | CH |
| NRAS | 1 | 115256530 | 115256530 | Missense | SNP | G | T | c.(181-183)Caa>Aaa | p.Q61K | 0.074074 | MMRF_1927_1_PB | Tumor |
| PDS5B | 13 | 33284244 | 33284244 | Splice site | SNP | G | A | c.e19+1 |  | 0.086957 | MMRF_2290_1_PB | CH |
| PIGT | 20 | 44049212 | 44049219 | Frameshift | DEL | TCAGGACG | - | c.(910-921)tatcaggacgtcfs | p.QDV305fs | 0.33460076 | MMRF_2652_1_PB | Germline |
| PPM1D | 17 | 58740726 | 58740727 | Frameshift | INS | - | C | c.(1630-1635)ggccccfs | p.GP544fs | 0.13438735 | MMRF_1186_2_PB | CH |
| PPM1D | 17 | 58740642 | 58740642 | Nonsense | SNP | C | A | c.(1546-1548)tCa>tAa | p.S516* | 0.034602 | MMRF_1641_1_PB | CH |
| PPM1D | 17 | 58740809 | 58740809 | Nonsense | SNP | C | T | c.(1714-1716)Cga>Tga | p.R572* | 0.02765 | MMRF_1722_1_PB | CH |
| PPM1D | 17 | 58740384 | 58740388 | Frameshift | DEL | TGAAT | - | c.(1288-1293)gtgaatfs | p.VN430fs | 0.05 | MMRF_1744_1_PB | CH |
| PPM1D | 17 | 58740467 | 58740467 | Nonsense | SNP | C | T | c.(1372-1374)Cga>Tga | p.R458* | 0.029851 | MMRF_2524_1_PB | CH |
| PPM1D | 17 | 58740479 | 58740479 | Nonsense | SNP | C | T | c.(1384-1386)Caa>Taa | p.Q462* | 0.022727 | MMRF_2562_1_PB | CH |
| PPM1D | 17 | 58740806 | 58740806 | Nonsense | SNP | C | T | c.(1711-1713)Cag>Tag | p.Q571* | 0.039526 | MMRF_2598_1_PB | CH |
| PPM1D | 17 | 58740546 | 58740546 | Nonsense | SNP | T | G | c.(1450-1452)tTa>tGa | p.L484* | 0.018868 | MMRF_2707_1_PB | Tumor |
| PRPF40B | 12 | 50031367 | 50031367 | Splice site | SNP | G | A | c.(1675-1677)Ggc>Agc | p.G559S | 0.046154 | MMRF_2580_1_PB | CH |
| PRPF8 | 17 | 1560055 | 1560055 | Splice site | SNP | A | G | c.(5506-5508)Ttg>Ctg | p.L1836L | 0.351648 | MMRF_2224_1_PB | Germline |
| RAD21 | 8 | 117861255 | 117861255 | Nonsense | SNP | G | C | c.(1633-1635)tCa>tGa | p.S545* | 0.070968 | MMRF_2245_1_PB | CH |
| SETD2 | 3 | 47165927 | 47165927 | Nonsense | SNP | C | A | c.(199-201)Gaa>Taa | p.E67* | 0.029091 | MMRF_1912_1_PB | Tumor |
| SF3B1 | 2 | 198266831 | 198266831 | Missense | SNP | C | A | c.(2101-2103)Gtt>Ttt | p.V701F | 0.083333 | MMRF_1542_1_PB | Tumor |
| SF3B1 | 2 | 198267360 | 198267360 | Missense | SNP | T | G | c.(1996-1998)aAg>aCg | p.K666T | 0.285714 | MMRF_2692_1_PB | Tumor |
| SF3B1 | 2 | 198267491 | 198267491 | Missense | SNP | C | G | c.(1864-1866)gaG>gaC | p.E622D | 0.042735 | MMRF_2742_1_PB | CH |
| SRSF2 | 17 | 74732959 | 74732959 | Missense | SNP | G | C | c.(283-285)cCc>cGc | p.P95R | 0.031496 | MMRF_1671_4_PB | CH |
| SRSF2 | 17 | 74732959 | 74732959 | Missense | SNP | G | T | c.(283-285)cCc>cAc | p.P95H | 0.072917 | MMRF_1760_1_PB | CH |
| SRSF2 | 17 | 74732959 | 74732959 | Missense | SNP | G | T | c.(283-285)cCc>cAc | p.P95H | 0.333333 | MMRF_2138_1_PB | CH |
| TERT | 5 | 1294312 | 1294312 | Missense | SNP | C | T | c.(688-690)cGa>cAa | p.R230Q | 0.041667 | MMRF_1489_1_PB | CH |
| TET2 | 4 | 106164934 | 106164934 | Splice site | SNP | G | T | c.(3802-3804)Gag>Tag | p.E1268* | 0.068966 | MMRF_1031_1_PB | CH |
| TET2 | 4 | 106197285 | 106197285 | Missense | SNP | T | C | c.(5617-5619)aTt>aCt | p.I1873T | 0.161538 | MMRF_1401_1_PB | CH |
| TET2 | 4 | 106164071 | 106164071 | Missense | SNP | C | T | c.(3580-3582)cCt>cTt | p.P1194L | 0.014706 | MMRF_1413_1_PB | CH |
| TET2 | 4 | 106157346 | 106157352 | Frameshift | DEL | AATAAAG | - | c.(2245-2253)caaataaagfs | p.QIK749fs | 0.03831418 | MMRF_1484_1_PB | CH |
| TET2 | 4 | 106162568 | 106162568 | Missense | SNP | G | C | c.(3481-3483)aGa>aCa | p.R1161T | 0.038055 | MMRF_1485_1_PB | CH |
| TET2 | 4 | 106196237 | 106196237 | Nonsense | SNP | C | T | c.(4570-4572)Cag>Tag | p.Q1524* | 0.047059 | MMRF_1510_1_PB | CH |
| TET2 | 4 | 106197309 | 106197309 | Missense | SNP | A | G | c.(5641-5643)cAt>cGt | p.H1881R | 0.112245 | MMRF_1511_1_PB | CH |
| TET2 | 4 | 106164068 | 106164068 | Missense | SNP | G | A | c.(3577-3579)tGt>tAt | p.C1193Y | 0.063158 | MMRF_1511_1_PB | CH |
| TET2 | 4 | 106158372 | 106158375 | Frameshift | DEL | ACCA | - | c.(3271-3276)acaccafs | p.TP1091fs | 0.16541353 | MMRF_1555_1_PB | CH |
| TET2 | 4 | 106190887 | 106190887 | Nonsense | SNP | C | T | c.(4165-4167)Cag>Tag | p.Q1389* | 0.067308 | MMRF_1725_1_PB | CH |
| TET2 | 4 | 106164897 | 106164897 | Nonsense | SNP | C | A | c.(3763-3765)taC>taA | p.Y1255* | 0.048649 | MMRF_1736_1_PB | CH |
| TET2 | 4 | 106162586 | 106162586 | Splice site | SNP | G | C | c.(3499-3501)aGg>aCg | p.R1167T | 0.333333 | MMRF_1740_1_PB | CH |
| TET2 | 4 | 106197365 | 106197365 | Missense | SNP | G | T | c.(5698-5700)Gtc>Ttc | p.V1900F | 0.056452 | MMRF_1760_1_PB | CH |
| TET2 | 4 | 106197606 | 106197606 | Missense | SNP | C | T | c.(5938-5940)aCa>aTa | p.T1980I | 0.434641 | MMRF_1781_1_PB | Germline |
| TET2 | 4 | 106193724 | 106193724 | Missense | SNP | T | C | c.(4186-4188)Tgc>Cgc | p.C1396R | 0.1875 | MMRF_1838_1_PB | CH |
| TET2 | 4 | 106193820 | 106193820 | Nonsense | SNP | G | T | c.(4282-4284)Gag>Tag | p.E1428* | 0.111111 | MMRF_1846_1_PB | CH |
| TET2 | 4 | 106162561 | 106162561 | Missense | SNP | G | C | c.(3475-3477)Gct>Cct | p.A1159P | 0.017964 | MMRF_1893_1_PB | CH |
| TET2 | 4 | 106164917 | 106164917 | Missense | SNP | G | T | c.(3784-3786)cGg>cTg | p.R1262L | 0.322115 | MMRF_1939_1_PB | CH |
| TET2 | 4 | 106155445 | 106155445 | Nonsense | SNP | C | T | c.(346-348)Caa>Taa | p.Q116* | 0.038961 | MMRF_2095_1_PB | CH |
| TET2 | 4 | 106194077 | 106194077 | Splice site | SNP | T | G | c.e10+2 |  | 0.029126 | MMRF_2137_1_PB | CH |
| TET2 | 4 | 106156316 | 106156317 | Frameshift | DEL | TT | - | c.(1216-1218)cttfs | p.L406fs | 0.30827068 | MMRF_2138_1_PB | CH |
| TET2 | 4 | 106155778 | 106155779 | Frameshift | INS | - | A | c.(679-681)gaafs | p.E227fs | 0.22641509 | MMRF_2138_1_PB | CH |
| TET2 | 4 | 106158436 | 106158436 | Missense | SNP | G | A | c.(3337-3339)Gat>Aat | p.D1113N | 0.458333 | MMRF_2286_1_PB | Germline |
| TET2 | 4 | 106197324 | 106197324 | Missense | SNP | T | C | c.(5656-5658)tTa>tCa | p.L1886S | 0.063291 | MMRF_2310_1_PB | CH |
| TET2 | 4 | 106197554 | 106197554 | Missense | SNP | A | G | c.(5887-5889)Act>Gct | p.T1963A | 0.465649 | MMRF_2335_1_PB | Germline |
| TET2 | 4 | 106190797 | 106190797 | Missense | SNP | C | T | c.(4075-4077)Cgt>Tgt | p.R1359C | 0.026316 | MMRF_2545_1_PB | CH |
| TET2 | 4 | 106196705 | 106196705 | Nonsense | SNP | C | T | c.(5038-5040)Cag>Tag | p.Q1680* | 0.4 | MMRF_2570_1_PB | Germline |
| TET2 | 4 | 106157684 | 106157685 | Frameshift | INS | - | G | c.(2584-2589)ttgcatfs | p.H863fs | 0.28104575 | MMRF_2637_1_PB | CH |
| TET2 | 4 | 106158530 | 106158530 | Missense | SNP | A | T | c.(3430-3432)gAg>gTg | p.E1144V | 0.48 | MMRF_2788_1_PB | Germline |
| TNFAIP3 | 6 | 138192621 | 138192628 | Frameshift | DEL | GTCGAGAA | - | c.(256-264)tgtcgagaafs | p.CRE86fs | 0.22077922 | MMRF_1897_1_PB | Tumor |
| TP53 | 17 | 7577114 | 7577114 | Missense | SNP | C | T | c.(823-825)tGt>tAt | p.C275Y | 0.068182 | MMRF_1024_2_PB | CH |
| TP53 | 17 | 7577077 | 7577077 | Missense | SNP | C | G | c.(859-861)gaG>gaC | p.E287D | 0.4875 | MMRF_1281_1_PB | Germline |
| TP53 | 17 | 7579414 | 7579414 | Nonsense | SNP | C | T | c.(271-273)tgG>tgA | p.W91* | 0.2 | MMRF_1339_1_PB | Tumor |
| TP53 | 17 | 7578190 | 7578190 | Missense | SNP | T | C | c.(658-660)tAt>tGt | p.Y220C | 0.032609 | MMRF_1339_1_PB | CH |
| TP53 | 17 | 7578242 | 7578242 | Missense | SNP | C | A | c.(607-609)Gtg>Ttg | p.V203L | 0.293413 | MMRF_1431_1_PB | CH |
| TP53 | 17 | 7578491 | 7578492 | Frameshift | INS | - | A | c.(436-441)tgggttfs | p.V147fs | 0.18032787 | MMRF_1871_1_PB | CH |
| TP53 | 17 | 7576911 | 7576911 | Missense | SNP | G | C | c.(934-936)aCc>aGc | p.T312S | 0.51087 | MMRF_2050_1_PB | Germline |
| TP53 | 17 | 7578457 | 7578457 | Missense | SNP | C | T | c.(472-474)cGc>cAc | p.R158H | 0.556522 | MMRF_2545_1_PB | Germline |
| TP53 | 17 | 7578404 | 7578404 | Missense | SNP | A | T | c.(526-528)Tgc>Agc | p.C176S | 0.031447 | MMRF_2690_1_PB | Tumor |
| TP53 | 17 | 7579353 | 7579353 | Missense | SNP | C | T | c.(334-336)Ggc>Agc | p.G112S | 0.463415 | MMRF_2711_1_PB | Germline |
| TP53 | 17 | 7578508 | 7578508 | Missense | SNP | C | G | c.(421-423)tGc>tCc | p.C141S | 0.480769 | MMRF_2721_1_PB | Germline |
| TP53 | 17 | 7573972 | 7573972 | Missense | SNP | T | A | c.(1054-1056)gAt>gTt | p.D352V | 0.465517 | MMRF_2847_1_PB | Germline |
| TRAF3 | 14 | 103371673 | 103371673 | Nonsense | SNP | G | A | c.(1258-1260)tGg>tAg | p.W420* | 0.032051 | MMRF_2739_1_PB | Tumor |
| U2AF1 | 21 | 44514777 | 44514777 | Missense | SNP | T | C | c.(469-471)cAg>cGg | p.Q157R | 0.164384 | MMRF_1033_1_PB | CH |
| U2AF1 | 21 | 44514777 | 44514777 | Missense | SNP | T | G | c.(469-471)cAg>cCg | p.Q157P | 0.309942 | MMRF_2064_2_PB | CH |
