# Supplementary material for: Clinical Outcomes and Evolution of Clonal Hematopoiesis in Patients with Newly Diagnosed Multiple Myeloma
Source: Cancer Res Commun. 2023 Dec 18;3(12):2560–71. doi: 10.1158/2767-9764.CRC-23-0093 (PMC10730502; doi:10.1158/2767-9764.CRC-23-0093)
Supplement: Supplementary Table 4 — Characteristics of the 8 patients who developed a second primary malignancy. [file crc-23-0093-s11.docx]

**Supplementary Table 4. Characteristics of the 8 patients who developed a second primary malignancy**

| **Patient** | **SPM** | **Day at time of SPM Diagnosis** | **Gender** | **Age at time of MM diagnosis** | **MM Subtype** | **Transplant** | **Lenalidomide maintenance** | **CH** |
| --- | --- | --- | --- | --- | --- | --- | --- | --- |
| MMRF_2076 | Chronic Myeloid Leukemia | 74 | Female | 72 | IgG Kappa | Yes | No | None |
| MMRF_1045 | Acute myeloid leukemia | 609 | Female | 81 | IgG Kappa | No | Yes | ASXL1 p.HHCHREAA630fs |
| MMRF_1797 | Myelodysplastic syndrome | 617 | Male | 64 | IgA Lambda | Yes | Yes | None |
| MMRF_1574 | Acute myeloid leukemia | 1577 | Male | 65 | IgG Kappa | Yes | Yes | None |
| MMRF_1881 | Acute myeloid leukemia | 1088 | Male | 66 | IgG Kappa | Yes | Yes | None |
| MMRF_2568 | Myelodysplastic syndrome | 723 | Male | 69 | Lambda light chain | Yes | Yes | None |
| MMRF_2225 | Acute myeloid leukemia | 639 | Female | 53 | IgA Lambda | Yes | No | None |
| MMRF_1706 | Myelodysplastic syndrome | 455 | Male | 66 | IgA Kappa | Yes | Yes | None |
| MMRF_1706 | Diffuse Large B cell Lymphoma | 1181 | Male | 66 | IgA Kappa | Yes | Yes | None |
