# Supplementary material for: Clinical Outcomes and Evolution of Clonal Hematopoiesis in Patients with Newly Diagnosed Multiple Myeloma
Source: Cancer Res Commun. 2023 Dec 18;3(12):2560–71. doi: 10.1158/2767-9764.CRC-23-0093 (PMC10730502; doi:10.1158/2767-9764.CRC-23-0093)
Supplement: Supplementary Table 5 — List of mutations classified as CH in the sequential samples. [file crc-23-0093-s12.docx]

**Supplementary Table 5. List of mutations classified as CH in the sequential samples**

| Gene | Chromosome | Start Position | End Position | Variant Classification | Variant Type | Reference Allele | Tumor Seq Allele2 | Codon Change | Protein Change | Allele Frequency | Sample |
| --- | --- | --- | --- | --- | --- | --- | --- | --- | --- | --- | --- |
| ASXL1 | 20 | 31022441 | 31022442 | Frameshift | INS | - | G | c.(2596-2598)gggfs | p.G866fs | 0.33769634 | MMRF_1210_2_PB |
| DNMT3A | 2 | 25470535 | 25470535 | Nonsense | SNP | C | T | c.(937-939)tgG>tgA | p.W313* | 0.210526 | MMRF_1671_4_PB |
| DNMT3A | 2 | 25470535 | 25470535 | Nonsense | SNP | C | T | c.(937-939)tgG>tgA | p.W313* | 0.142138 | MMRF_1671_5_PB |
| DNMT3A | 2 | 25471016 | 25471016 | Nonsense | SNP | G | A | c.(745-747)Cag>Tag | p.Q249* | 0.040404 | MMRF_1739_2_PB |
| DNMT3A | 2 | 25470536 | 25470536 | Nonsense | SNP | C | T | c.(937-939)tgG>tgA | p.W313* | 0.037613 | MMRF_2412_2_PB |
| DNMT3A | 2 | 25468888 | 25468888 | Splice site | SNP | C | A | c.e12+1 |  | 0.048611 | MMRF_1079_4_PB |
| DNMT3A | 2 | 25468888 | 25468888 | Splice site | SNP | C | A | c.e12+1 |  | 0.038043 | MMRF_1079_6_PB |
| DNMT3A | 2 | 25461998 | 25461998 | Splice site | SNP | C | T | c.e22+1 |  | 0.103641 | MMRF_2059_2_PB |
| DNMT3A | 2 | 25463262 | 25463262 | Frameshift | DEL | T | - | c.(2230-2232)aagfs | p.K744fs | 0.13907285 | MMRF_1628_2_PB |
| DNMT3A | 2 | 25458595 | 25458595 | Missense | SNP | A | G | c.(2578-2580)Tgg>Cgg | p.W860R | 0.072464 | MMRF_1030_3_PB |
| DNMT3A | 2 | 25458595 | 25458595 | Missense | SNP | A | G | c.(2578-2580)Tgg>Cgg | p.W860R | 0.122283 | MMRF_1030_4_PB |
| GATA1 | X | 48650568 | 48650568 | Missense | SNP | C | G | c.(538-540)Ctc>Gtc | p.L180V | 0.058252 | MMRF_1624_3_PB |
| NF1 | 17 | 29679299 | 29679299 | Nonsense | SNP | G | A | c.(7480-7482)tgG>tgA | p.W2494* | 0.033835 | MMRF_2111_2_PB |
| NRAS | 1 | 115256528 | 115256528 | Missense | SNP | T | A | c.(181-183)caA>caT | p.Q61H | 0.049505 | MMRF_1269_3_PB |
| RAD21 | 8 | 117864947 | 117864947 | Splice site | SNP | G | T | c.(1162-1164)Ctc>Atc | p.L388I | 0.416667 | MMRF_1795_2_PB |
| SF3B1 | 2 | 198267360 | 198267360 | Missense | SNP | T | G | c.(1996-1998)aAg>aCg | p.K666T | 0.040404 | MMRF_1957_2_PB |
| TET2 | 4 | 106164897 | 106164897 | Nonsense | SNP | C | A | c.(3763-3765)taC>taA | p.Y1255* | 0.142857 | MMRF_1736_2_PB |
| TET2 | 4 | 106197405 | 106197405 | Missense | SNP | G | A | c.(5737-5739)gGc>gAc | p.G1913D | 0.02924 | MMRF_2301_2_PB |
| TP53 | 17 | 7577539 | 7577539 | Missense | SNP | G | A | c.(742-744)Cgg>Tgg | p.R248W | 0.026549 | MMRF_1269_3_PB |
| U2AF1 | 21 | 44514777 | 44514777 | Missense | SNP | T | G | c.(469-471)cAg>cCg | p.Q157P | 0.309942 | MMRF_2064_2_PB |
