# Supplementary material for: Clinical Outcomes and Evolution of Clonal Hematopoiesis in Patients with Newly Diagnosed Multiple Myeloma
Source: Cancer Res Commun. 2023 Dec 18;3(12):2560–71. doi: 10.1158/2767-9764.CRC-23-0093 (PMC10730502; doi:10.1158/2767-9764.CRC-23-0093)
Supplement: Supplementary Table 6 — Temporal changes in CH clones in 13 patients with sequential samples. [file crc-23-0093-s13.docx]

**Supplementary Table 6. Temporal changes in CH clones in 13 patients with sequential samples**

|  | **Time Point 1** | | | | **Time Point 2** | | | |
| --- | --- | --- | --- | --- | --- | --- | --- | --- |
| **Sample** | Gene | VAF | Protein Change | cDNA Change | Gene | VAF | Protein Change | cDNA Change |
| MMRF_1671 | DNMT3A | 0.09816 | p.W90* | c.270G>A | DNMT3A | 0.210526 | p.W90* | c.270G>A |
| MMRF_1030 | None |  |  |  | DNMT3A | 0.072464 | p.W637R | c.1909T>C |
| MMRF_1079 | DNMT3A | 0.164384 | splice |  | DNMT3A | 0.048611 | splice |  |
| MMRF_1210 | None |  |  |  | ASXL1 | 0.33769634 | p.G643fs |  |
| MMRF_1624 | None |  |  |  | GATA1 | 0.058252 | p.L180V | c.538C>G |
| MMRF_1628 | None |  |  |  | DNMT3A | 0.13907285 | p.K521fs | c.1562delA |
|  |  |  |  |  | SRSF2 | 0.031496 | p.P95R | c.284C>G |
| MMRF_1736 | TET2 | 0.048649 | p.Y1255* | c.3765C>A | TET2 | 0.142857 | p.Y1255* | c.3765C>A |
| MMRF_1739 | None |  |  |  | DNMT3A | 0.040404 | p.Q26* | c.76C>T |
| MMRF_1957 | None |  |  |  | SF3B1 | 0.040404 | p.K666T | c.1997A>C |
| MMRF_2059 | None |  |  |  | DNMT3A | 0.103641 | splice |  |
| MMRF_2111 | None |  |  |  | NF1 | 0.033835 | p.W2494* | c.7482G>A |
| MMRF_2301 | None |  |  |  | TET2 | 0.02924 | p.G1913D | c.5738G>A |
| MMRF_2412 | None |  |  |  | DNMT3A | 0.037613 | p.W90* | c.269G>A |
